# Evaluation and characterization of indigenous rice (*Oryza sativa* L.) landraces resistant to brown planthopper *Nilaparvata lugens* (St*å*l.) biotype 4

**DOI:** 10.7717/peerj.14360

**Published:** 2022-11-04

**Authors:** Debashis Roy, Abhisek Biswas, Sukamal Sarkar, Gautam Chakraborty, Ahmed Gaber, Mohamed I. Kobeasy, Akbar Hossain

**Affiliations:** 1 Department of Agricultural Entomology, Bidhan Chandra Krishi Viswavidyalaya, Nadia, West Bengal, India; 2 Plant Protection, Dhaanya Ganga Krishi Vigyan Kendra, Ramakrishna Mission Vivekananda Educational and Research Institute, Murshidabad, West Bengal, India; 3 Department of Agricultural and Environmental Sciences (DiSAA), University of Milan, Milan, Italy; 4 School of Agriculture and Rural Development, Ramakrishna Mission Vivekananda Educational and Research Institute, Narendrapur, Kolkata, West Bengal, India; 5 Department of Biology, College of Science, Taif University, Taif, Saudi Arabia; 6 Department of Chemistry, College of Science, Taif University, Taif, Saudi Arabia; 7 Department of Agronomy, Bangladesh Wheat and Maize Research Institute, Dinajpur, Rangpur, Bangladesh

**Keywords:** Antixenosis, Antibiosis, Brown planthopper, Plant defence, Folk rice, Principal component, Correlation, Cluster dendrogram

## Abstract

Evaluation and identification of resistant donors for brown planthopper (BPH) *Nilaparvata lugens* (St*å*l.), an economically important insect pest of rice, is a continuous process to develop new resistant rice varieties. However, several rice landraces of north-eastern India are not yet characterized for BPH resistance. In the present study, a set of 218 rice landraces were screened in both greenhouse and open-field conditions for three consecutive years, and thereafter forty selected promising entries were explored to evaluate their phenotypic and genotypic reactions against BPH biotype 4. Based on phenotypic evaluations, five landraces were identified as resistant, while 31 were moderately resistant, and grouped under the major cluster I and II, respectively, in a circular dendrogram. Antixenosis and antibiosis studies of these landraces divulged that, compared to the susceptible check variety, resistant landraces exhibited the lowest feeding rate, survival, and nymphal and adult settling, but higher frequency of unhatched eggs of BPH. Un-infested resistant landraces registered higher levels of ascorbic acid, oxalic acid and crude silica, however, elevated levels of total free amino acid, potassium and crude silica were observed under BPH herbivory. The present study focuses on identifying new donors having BPH resistance resources which could be useful in genomic studies for the development of BPH biotype 4 resistant rice varieties.

## Introduction

Rice (*Oryza sativa* L.) is consumed by more than one-third of the total human population across the globe as a major source of carbohydrates among cereal crops (Fitzgerald, McCouch & Hall, 2009; Jena et al., 2015). The brown planthopper (BPH), *Nilaparvata lugens* (St*å*l) (Hemiptera: Delphacidae), is one of the economically important insect pests identified in this crop, having an international significance (Normile, 2008; Heong & Hardy, 2009). This phloem sap feeder transmits *rice ragged stunt virus* (RRSV) and *rice grassy stunt virus* (RGSV), and holds the ability to cause more than 60% economic yield loss under favourable environmental conditions throughout Asia (Kumar, Maurya & Tiwari, 2012; Wei et al., 2019). Several chemical insecticides are registered to control rice BPH, but the unscientific and injudicious application of those products breaks the natural pest defender ratio in the field (Sarao & Mangat, 2014; Roy & Chakraborty, 2022). Therefore, the development of new varieties resistant to BPH is extremely important. The identification of new sources of resistance from landraces, wild cultivars or germplasms enables the plant breeders to amplify the resistance breeding program through genetic modification (Guo, Liao & Chuang, 2019). Modern high yielding varieties are primarily designed to meet the economic expectations of the farming communities and the demand of the rising populations, but these types do not have biotic stress resistance, especially for BPH. In several provinces of India where *N. lugens* outbreak occurs often (Andhra Pradesh, Odisha, Delhi, West Bengal and Punjab), this lack of resistance caused complete crop loss (Anant et al., 2021). Thus, in order to generate promising cultivars that provide persistent and targeted resistance to field populations of BPH, it is therefore required to find novel *N. lugens* resistance mechanisms. In comparison to developed varieties, folk rice or indigenous landraces have a diverse resistance features. The eastern and north-eastern provinces of India, known to have highly diverse rice landraces belonging to this region are expected to have high genetic variability for various pests and diseases including the BPH (Sinha, Mallick & Mishra, 2015; Anant et al., 2021; Babu et al., 2022). West Bengal, an eastern province of India, possesses many tribal belts. These tribal zones harbour many indigenous rice landraces, and well known to be one of the primary centres of origin of rice (Karmakar et al., 2012; Adak et al., 2020). However, evaluation and systematic exploration of most of the West Bengal rice landraces for their true genetic potential, and their characterization for BPH resistance were not yet done broadly.

The biophysical factors of a plant interfere with the feeding, orientation, mating or oviposition mechanisms of insects. In contrast, the biochemical factors are either the primary nutrients or secondary non-nutritional chemicals of plants that affect insect biology. Some of these non-nutritional chemicals are associated with feeding deterrence, repellence or toxicity on insects (Saxena, 1986). However, the potential nutritive factors of a plant also play a pivotal role in enhancing its resistance to insects, even in the absence of these chemicals (Mitchell et al., 2016). Host plant shows varied kind of reactions upon feeding and infesting by insects, and alteration of nutritional biochemistry of a plant also takes place in this response (Vanitha, Suresh & Gunathilagaraj, 2011). Thus, a strong basis for developing resistant varieties against BPH biotype 4 should be aligned towards ascertaining the resistance by imparting bio-chemicals and genetic resources and applying those as cues in the resistance breeding program.

In the present study, systematic phenotyping of traditional rice landraces of West Bengal was performed for BPH resistance over a continuous span of 3 years. Based on the phenotypic data, 40 promising landraces were further examined for antixenosis, antibiosis, and biochemical parameters concerning their host-plant resistance.

## Materials and Methods

### Plant and insect material

The present study was undertaken with a total of 218 rice landraces, native to different provinces of north-east India, previously registered under West Bengal Biodiversity Board, Department of Environment, Government of West Bengal, Salt Lake, Kolkata–700106, India (Table S1). The resistant Ptb33 and susceptible variety Swarna (MTU 7029) were considered as a positive and negative control, respectively, collected from National Rice Research Institute, Cuttack, Odisha-India germplasm unit. The insect culture of BPH biotype 4 was obtained from the Department of Zoology, University of Burdwan, West Bengal, India in 2017 and thereafter maintained in the insect-proof glasshouse of Bidhan Chandra Krishi Viswavidyalaya (22.9452° N, 88.5336° E), Nadia, West Bengal, India under the controlled conditions of 28 ± 2 °C, 75 ± 5% relative humidity and a photoperiod of 14:10 h (light: dark). Mass rearing was done on 30 days old rice plants in wooden cages of 120 × 75 × 150 cm dimensions having separate compartments tunicated with fine wire mesh on all sides. Fifteen to twenty adult gravid female BPH were collected with an aspirator and released on sowed plants of Swarna, previously placed in oviposition chamber. After 2 days of egg laying, the gravid females were recollected and shifted on another fresh set of Swarna plants for further oviposition. Plants containing eggs were taken out of cages with entire trays and shifted in another separate compartment for hatching of first instar nymphs. A new set of plants were provided inside the cubicle of nymphs according to the date of exposure of eggs. Similarly, adults were also maintained after emergence in the same or separate compartment as and when required. Both the nymphs of desirable instars and adults were used by culling from the specific compartments as per the various experimental quests. Likewise, a continuous pure mother culture of BPH was sustained throughout the period of investigation.

### Phenotyping for BPH resistance

Primary screening of 218 rice landraces for BPH biotype 4 resistance was conducted under greenhouse and open-field conditions in 2017–2019. Cluster analysis of 218 rice landraces and two control varieties was done based on the phenotypic scores and two quantitative parameters, such as the number of BPH per three plants and percent chaffy grains. The similarity matrix was generated through the simple Euclidean distance across all parameters of different landraces, and this matrix was used in a hierarchical clustering technique of Ward’s minimum variance method using ‘R’ software, version 4.0.2 (R Core Team, 2020).

### Greenhouse screening

The standard seedbox screening method of IRRI (2002) with suitable modifications by Jena et al. (2006) was followed for the free-choice test to evaluate BPH resistance to 218 rice landraces in a complete randomized design and replicated thrice. At the seedling three-leaf stage in the screening trays, 2^nd^ instar nymphs of BPH in the rearing cages were released artificially onto the seedlings by visually ensuring the infestation of each seedling with at least 8–10 nymphs and were monitored at a regular interval for plant damage by BPH. When Swarna plants on one side exhibited intense damage, the entire cage was rotated by 180° for equal reaction on both sides. On the other hand, the ‘isolated cage-test method’ was followed by taking individual plastic pots (D × H, 8 × 20 cm) for each landrace to conduct no-choice screening (De Vos & Jander, 2008). Twenty freshly germinated seeds of each landrace were individually seeded concentrically in a single pot including Ptb33 and Swarna and encircled with a transparent OHP sheet-made hollow cylindrical structure (D × H, 6 × 30 cm), roofed with 80-mesh insect-proof net pieces at the top. Like the previous experiment, 2^nd^ instar BPH nymphs were artificially released from the top by ensuring at least 35–40 individuals per pot. Both the experiments were terminated after the complete wilting of more than 90% of Swarna seedlings, and the damage to all landraces was computed individually. The score was taken based on 0–9 scale according to the international standard evaluation system (Horgan et al., 2015).

### Open-field screening

Approximately 45–50 pre-soaked germinated seeds of each landrace, including both Ptb33 and Swarna, were individually sown into 1 m^2^ plot at a BPH biotype 4 hotspot (Burdwan district of West Bengal, India; 23.2324° N, 87.8615° E) (Krishnaiah & Varma, 2012). The plots were selected in a randomized complete block design throughout the field and replicated thrice (Bhogadhi & Bentur, 2015). Sowing was done deliberately late in the 2^nd^ fortnight of July with a closer spacing (r-r × p-p, 15 × 15 cm) to get a maximum infestation of BPH (Satpathi, Chakraborty & Acharjee, 2012). Manual weeding operation was done at 25 and 45 days after transplanting (DAT) and a 15 cm water level was maintained followed by recommended agronomic practices except plant protection measures for standard BPH multiplication in the field. Phenotypic scoring was done by taking randomly selected 20 plants per replicated plot according to the damage scale 0–9 when Swarna plants exhibited ‘hopper burn’ symptoms (Sai Harini et al., 2013). Mean numbers of BPH nymphs and adults per three plants and chaffy grains (%) were also enumerated by following Timmanagouda & Maheswaran (2017).

### Biophysical and biochemical study of rice landraces

The evaluation of different biophysical and biochemical parameters related to BPH resistance was conducted in a set of three replications with 40 promising rice landraces (see ‘Results’ section), selected from the three years phenotyping scores, in 2020–2021. Biophysical studies were conducted in a separate compartment of the insect-proof glasshouse of Bidhan Chandra Krishi Viswavidyalaya, whereas biochemical studies were carried out at the Indian Institute of Science Education and Research (IISER) Kolkata, West Bengal, India (22.9638° N, 88.5245° E). To determine the biochemical components, seeds were sown separately in two plastic containers for each tested landrace with no additional nutrient, and one set of 30 day-old seedlings was infested with 2^nd^–3^rd^ instar BPH nymphs for 7 days. The green leaf sheaths of both healthy and BPH infested plants were used for the analysis of total phenol (TP), reducing sugar (RS), ascorbic acid (AS), oxalic acid (OA), crude silica (CS) and total free amino acid (TFA), while nitrogen (N), phosphorus (P) and potassium (K) were estimated from oven-dried (60 °C for 72 h) and ground plant materials.

### Excretion of honeydew

Evaluation on the honeydew excretion of BPH was conducted with 30 day-old potted seedlings by following Sogawa & Pathak (1970), as described in our previous study (Roy et al., 2021). Five numbers of each 1-day-old adult females and 2^nd^ instar nymphs were placed separately to the lower stem portion of the 30 day-old potted seedling with an orange coloured bromocresol green treated filter paper around the base and an inverted and basal perforated transparent plastic cup (80 ml volume) on the filter paper incarcerating the insects to the stem portion of about 9 cm long. The hole of the cup was closed with a ball of non-absorbent cotton and the honeydew droplets excreted by BPH were turned into blue spots when they came in contact with the filter paper after 48 h of insect imprisonment. The area marked with blue colour was measured on a millimetre squared (mm^2^) graph paper sheet as the extent of feeding and also interpreted statistically.

### Settling behaviour

This experiment was carried out by taking 40 selected rice landraces seeded at random rows (10 seeds per row), 3.0 cm apart in a seedbox by following the standard procedure given by Sarao & Bentur (2016) as described in our previous study (Roy et al., 2021). The Swarna seeds were sown in two border rows, whereas a single row of Ptb33 was introduced in the middle of the box. The 2^nd^–3^rd^ instar BPH nymphs with at least 12–15 individuals per seedling were introduced on 15 day-old rice landraces and the experiment tray was covered immediately with an insect-proof cage. Data on the number of nymphs settled on each seedling was recorded from randomly selected five plants in each row at 1, 3 and 5 days after release. In a parallel set of experiments, around 800 pairs of adults were released onto the 30 day-old seedlings, previously grown in a well puddled soil-filled tray, with the help of a giant aspirator under a free-choice fashion and similarly covered with an insect-proof cage. Numbers of adult males and females alighting on different landraces were visually counted at 6, 12, 24, 48, 72 and 96 h after release. The seedlings were manually disturbed after each observation in both cases for proper reorientation of the BPH nymphs and adults.

### Nymphal survival

The experiment on nymphal survivability was carried out by caging 1-day-old freshly hatched 1^st^ instar BPH nymphs on 15 day-old seedlings (20 nymphs per plant and replicated thrice) of all the landraces separately with fine muslin cloth ventilation (700,000 nanometer) along with Ptb33 and Swarna (Jena et al., 2015). Monitoring of the seedlings was done regularly for a consecutive 18 days, and the numbers of newly emerged adults were recorded and carefully removed from the seedlings. The percent of nymphal survival was enumerated using the formula of Heinrichs, Medrano & Rapusas (1985).

(1) $${\rm Percent\; }\left( {\rm \% } \right){\rm \; nymphal\; survival} = {\rm \; }\displaystyle{{{\rm number\; of\; adults\; emerged}} \over {{\rm number\; of\; nymphs\; released}}} \times 100$$

### Ovicidal test

Like the previous experiment, one mating pair of BPH adults (3-day-old) was confined to each tested rice seedling under a fine muslin ventilated cloth (700,000 nanometer), and replicated thrice. After 7 days the adults were removed and all the seedlings were observed for the hatching of nymphs from the day onward. The number of newly hatched nymphs was recorded and carefully removed from the plant using an aspirator. Seedlings were collected when nymphs stopped coming out after 15–18 days, and thereafter dissected under a stereoscopic zoom binocular microscope (40× magnifications) to examine the number of egg masses and the number of unhatched eggs. A total number of eggs were assumed to be the sum of the number of nymphs counted and the number of unhatched eggs. The percent unhatched eggs was enumerated by using the formula of Khan & Saxena (1985).

(2) $$\eqalign{& {\rm Percent\; }\left( {\rm \% } \right){\rm \; unhatched\; eggs} \cr&= {\rm \; }\displaystyle{{{\rm number\; of\; unhatched\; eggs}} \over {\left( {{\rm number\; of\; nymphs\; emerged\; } + {\rm number\; of\; unhatched\; eggs}} \right)}} \times 100}$$

### Estimation of TP, RS and AS

The quantity of TP, RS and AS in healthy and BPH infested rice landraces were estimated by a calorimetric assay using a spectrophotometer (UV-1900; Shimadzu, Kyoto, Japan), dinitrosalicylic acid reagent (DNS) method and volumetric method, respectively, described by Sadasivam & Manickam (2008).

(3) $$10{\rm \; ml\; contain} = {\rm \; }\displaystyle{{{\rm x\; } \times 10{\rm \; mg\; of\; glucose}} \over {0.1}} = {\rm \% \; of\; reducing\; sugar}$$

Absorbance corresponding to 0.1 ml of test sample = x mg of glucose.

(4) $$\eqalign{& {\rm Quantity\; of\; ascorbic\; acid\; }\left( {{\rm mg\; per\; }100{\rm \; g\; sample}} \right) \cr&= {\rm \; }\displaystyle{{0.5{\rm \; mg\; } \times {\rm V}2{\rm \; } \times 100{\rm \; ml\; } \times 100} \over {{\rm V}1{\rm \; ml\; } \times 5{\rm \; ml\; } \times {\rm Wt}.{\rm \; of\; the\; sample}}}}$$

where, V1 = known volume and V2 = titrated volume.

### Estimation of OA, CS and TFA

Quantitative estimation of OA in healthy and BPH infested rice landraces was done by a direct calorimetric method with Indole reagent (Bergerman & Elliot, 1955), whereas the spectrophotometric method (UV-1900; Shimadzu, Kyoto, Japan) was used to estimate the CS (Wei-min et al., 2005) and TFA content (Moore & Stein, 1948).

### Estimation of N, P and K

One gram each of oven-dried plant sample was taken from both healthy and BPH infested plants and N was estimated on a whole plant basis using the standard micro Kjeldahl method according to Piper (1966). For P and K, 250 mg of plant material was digested by wet digestion method (Piper, 1966) using a tri-acid mixture (nitric, sulphuric and perchloric acids in a 9:2:1 ratio) and the values were estimated with the help of a Systonics Digital Flame Photometer (Model S-931).

### Statistical analysis

The data obtained from different experiments related to biophysical and biochemical parameters were analyzed using analysis of variance (ANOVA) with the help of IRRISTAT 4.0 software (Sarao & Bentur, 2016). Data were transformed using arcsine and square root transformations before being subjected to statistical analysis whenever required. Tukey’s HSD test (*p* ≤ 0.05) was done using SPSS software (version 18.0: Inc., Chicago, IL, USA) to compare the significance of statistics in the activities of each observed biophysical and biochemical parameters among the tested rice landraces. Pairwise correlation by Pearson’s correlation method and Principal Component Analysis (PCA) was employed to establish a relationship among different BPH resistance traits in the tested rice landraces using XL-Stats 2020 software at https://www.xlstat.com/en/ (accessed on 18.05.2022).

## Results

### Mass screening

**Figure 1:**
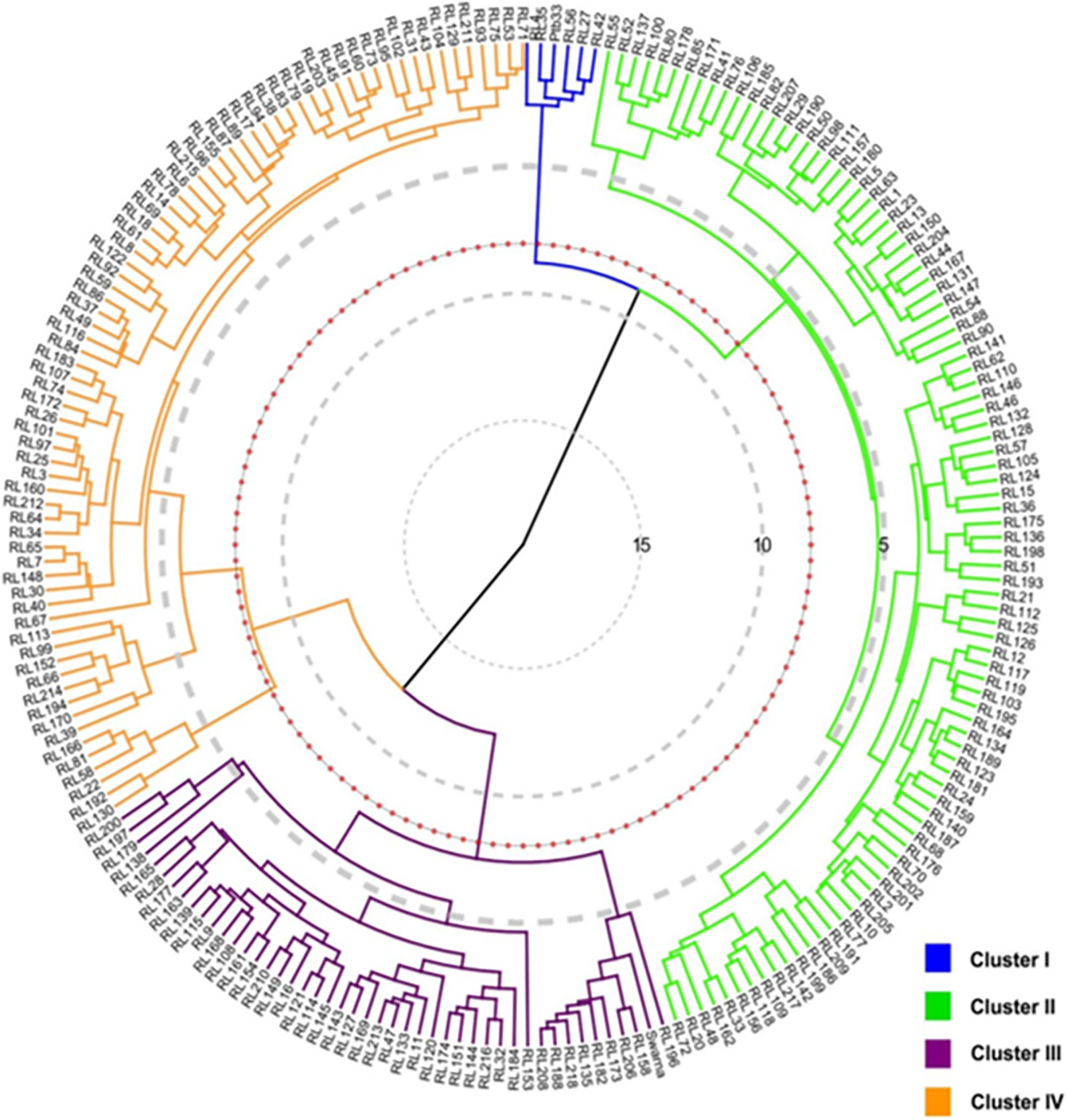
Circular cluster dendrogram based on similarity matrix enumerated from 218 rice landraces (RL) and 2 control (Ptb33 and Swarna) varieties.

The resistance scores were observed among 218 rice landraces ranged between 1.2 and 9.0 (greenhouse) and 1.1 and 9.0 (open-field), indicating a wide variation (Table S1). The five landraces *viz*. RL4, RL27, RL35, RL42 and RL56 were observed as resistant against BPH and grouped under the major cluster I, whereas most of the moderately resistant landraces were classified under cluster II (Fig. 1). Cluster III comprised 46 landraces closest to Swarna in the similarity matrix, however, the majority of moderately susceptible landraces constituted two sub-clusters under the major cluster IV.

### Antixenosis and antibiosis study

#### Honeydew excretion

The quantity of honeydew excreted by BPH nymphs varied significantly among the tested landraces (Table 1). The lowest honeydew for BPH nymphs was recorded in RL35 (27.9 mm^2^), followed by RL4 (30.3 mm^2^), RL42 (30.7 mm^2^) and RL27 (33.3 mm^2^), respectively, and were at par to Ptb33. A similar trend of honeydew excretion was also observed for 1-day-old adult BPH females (Table 1).

**Table 1:** Phenotypic reactions of selected rice landraces to BPH.

Type	Overall remarks	Area of honeydew (mm^2^)		Settling behaviour			Nymphal survival (%)	Unhatched eggs (%)
		Nymph	Adult female	Nymph	Adult male	Adult female		
RL4	R	30.3 ± 0.12^†^a	47.5 ± 0.37a	4.2 ± 0.03a	5.4 ± 0.09a	6.5 ± 0.05ab	25.6 (30.40)^‡^a	77.3 (62.58)c
RL27	R	33.3 ± 0.25a	59.4 ± 0.32a	6.4 ± 0.09a	5.7 ± 0.05a	6.8 ± 0.05ab	29.2 (32.71)a	84.1 (65.96)c
RL35	R	27.9 ± 0.28a	46.2 ± 0.24a	4.3 ± 0.06a	3.9 ± 0.06a	4.9 ± 0.05a	26.9 (30.40)a	70.8 (57.61)bc
RL42	R	30.7 ± 0.36a	49.1 ± 0.28a	3.2 ± 0.08a	3.0 ± 0.03a	5.9 ± 0.03ab	28.3 (32.96)a	81.2 (64.30)c
RL56	R	38.1 ± 0.71a	62.2 ± 0.48a	3.9 ± 0.03a	6.1 ± 0.05ab	4.8 ± 0.03a	32.7 (34.57)a	71.9 (57.99)bc
RL1	MR	37.3 ± 0.23a	69.5 ± 0.46a	9.2 ± 0.08ab	4.3 ± 0.05a	6.2 ± 0.04ab	41.3 (39.98)ab	72.2 (58.18)bc
RL190	MR	39.5 ± 0.53a	65.2 ±0.31a	6.2 ± 0.04a	6.5 ± 0.08ab	5.8 ± 0.07ab	46.2 (42.82)ab	68.7 (55.98)b
RL176	MR	34.9 ± 0.28a	81.2 ± 0.20a	5.1 ± 0.03a	4.3 ± 0.05a	6.8 ± 0.03ab	30.1 (33.27)a	53.5 (47.01)ab
RL23	MR	43.8 ± 0.18ab	72.5 ± 0.44a	4.6 ± 0.04a	4.6 ± 0.03a	5.6 ± 0.04ab	41.2 (39.93)ab	71.5 (57.73)bc
RL204	MR	40.1 ± 0.19a	65.8 ± 0.27a	5.6 ± 0.04a	3.6 ± 0.04a	4.9 ± 0.02a	32.8 (34.94)a	76.2 (60.80)c
RL54	MR	37.3 ± 0.23a	69.1 ± 0.33a	7.1 ± 0.10ab	4.1 ± 0.04a	5.8 ± 0.05ab	35.8 (36.75)ab	72.3 (58.24)bc
RL63	MR	40.1 ± 0.29a	64.2 ± 0.25a	7.3 ± 0.05ab	3.9 ± 0.04a	6.8 ± 0.07ab	27.9 (31.88)a	72.5 (58.37)bc
RL136	MR	38.2 ± 1.10a	67.2 ± 0.59a	4.3 ± 0.03a	4.1 ± 0.05a	6.2 ± 0.04ab	27.1 (31.37)a	58.6 (49.95)b
RL5	MR	63.5 ± 0.37ab	114.2 ± 0.79ab	7.3 ± 0.05ab	10.2 ± 0.14b	10.1 ± 0.09b	52.2 (46.26)ab	43.2 (41.09)ab
RL13	MR	70.7 ± 0.16b	138.3 ± 0.87ab	6.2 ± 0.06a	8.5 ± 0.06b	7.3 ± 0.05ab	36.9 (37.41)ab	49.2 (44.54)ab
RL192	MR	56.1 ± 0.71ab	176.2 ± 0.62b	8.6 ± 0.09ab	7.2 ± 0.05ab	7.3 ± 0.06ab	34.6 (36.03)ab	47.5 (43.57)ab
RL22	MR	61.1 ± 0.33ab	110.6 ± 1.12ab	8.9 ± 0.13ab	9.2 ± 0.16ab	10.2 ± 0.09b	42.2 (40.51)ab	49.4 (44.66)ab
RL166	MR	60.3 ± 0.25ab	120.8 ± 0.74ab	10.1 ± 0.08ab	8.1 ± 0.04b	9.5 ± 0.12b	54.9 (47.81)ab	50.2 (45.12)ab
RL58	MR	77.2 ± 0.41b	112.5 ± 0.41ab	7.3 ± 0.10ab	4.3 ± 0.04a	6.5 ± 0.03ab	34.2 (35.79)ab	58.4 (49.84)b
RL150	MR	49.5 ± 0.82ab	127.2 ± 0.39ab	7.6 ± 0.04ab	6.5 ± 0.06ab	8.6 ± 0.09b	43.2 (41.09)ab	54.9 (47.81)ab
RL44	MR	92.2 ± 0.53c	138.9 ± 0.72ab	10.1 ± 0.17ab	7.8 ± 0.11ab	11.2 ± 0.09b	31.2 (33.96)a	53.5 (47.01)ab
RL125	MR	56.2 ± 0.27ab	122.3 ± 0.66ab	6.2 ± 0.06a	7.1 ± 0.06ab	8.9 ± 0.14b	40.1 (39.29)ab	51.2 (45.69)ab
RL72	MR	50.1 ±0.32ab	112.3 ± 0.56ab	9.3 ± 0.05ab	4.2 ±0.03a	5.1 ± 0.03a	29.1 (32.64)a	58.2 (49.72)b
RL130	MR	49.0 ± 0.68ab	154.8 ± 1.17ab	7.0 ± 0.09ab	5.6 ± 0.07a	7.3 ± 0.07ab	42.8 (40.86)ab	49.0 (44.43)ab
RL81	MR	61.9 ± 0.18ab	161.3 ± 0.52b	9.2 ± 0.17ab	5.6 ± 0.06a	8.9 ± 0.04b	57.2 (49.14)ab	47.2 (43.39)ab
RL88	MR	59.1 ± 0.53ab	144.6 ± 0.82ab	9.3 ± 0.05ab	8.1 ± 0.10b	10.3 ± 0.08b	51.5 (45.86)ab	51.3 (45.74)ab
RL90	MR	62.9 ± 0.35ab	150.2 ± 1.41ab	7.3 ± 0.17ab	6.8 ± 0.08ab	8.2 ± 0.10b	29.1 (32.65)a	58.5 (49.89)b
RL110	MR	76.1 ± 0.51b	120.9 ± 0.74ab	8.1 ± 0.07ab	8.3 ± 0.09b	12.1 ± 0.09bc	45.8 (42.59)ab	55.6 (48.22)ab
RL186	MR	135.9 ± 0.69cd	190.2 ± 1.15b	12.3 ± 0.11b	10.1 ±0.04b	8.2 ± 0.07b	72.8 (58.56)b	38.2 (38.17)ab
RL199	MR	114.3 ± 1.06c	176.5 ± 0.94b	9.6 ± 0.10ab	7.6 ± 0.09ab	7.8 ± 0.08ab	58.1 (49.66)ab	39.6 (38.99)ab
RL20	MR	110.1 ± 0.82c	234.5 ± 0.89c	7.5 ± 0.05ab	6.5 ± 0.10ab	12.7 ± 0.15bc	52.1 (46.20)ab	42.2 (40.51)ab
RL118	MR	95.2 ± 0.40c	205.2 ± 1.26c	14.1 ± 0.11b	10.1 ± 0.09b	11.2 ± 0.07b	56.6 (48.79)ab	32.3 (34.63)a
RL33	MR	122.2 ± 0.46c	197.6 ± 0.62bc	9.6 ± 0.12ab	9.3 ± 0.16b	10.1 ± 0.06b	69.8 (56.66)b	38.2 (38.17)ab
RL162	MR	111.7 ± 1.14c	208.2 ± 1.52c	7.6 ± 0.03ab	11.2 ± 0.19b	10.2 ± 0.17b	53.6 (47.06)ab	31.2 (33.96)a
RL48	MR	109.7 ± 0.76c	238.7 ± 0.95c	10.5 ± 0.14b	8.1 ± 0.10b	12.2 ± 0.07bc	42.3 (40.57)ab	42.2 (40.51)ab
RL209	MR	119.2 ± 0.46c	224.0 ± 1.30c	12.5 ± 0.06b	9.5 ± 0.14b	11.6 ± 0.06b	75.8 (60.53)b	35.6 (36.63)a
RL156	MS	110.2 ± 0.56c	197.5 ± 1.28bc	13.2 ± 0.08b	10.1 ± 0.08b	8.2 ± 0.13b	81.1 (64.23)b	44.3 (41.73)ab
RL217	MS	129.6 ± 1.27cd	196.2 ± 0.67bc	12.1 ± 0.15b	10.1 ± 0.08b	9.1 ± 0.08b	76.6 (61.07)b	37.6 (37.82)ab
RL109	MS	114.5 ± 0.29c	210.9 ± 0.39c	10.1 ± 0.09ab	9.3 ± 0.17b	7.5 ± 0.07ab	51.2 (45.69)ab	35.4 (36.51)a
RL142	MS	127.9 ± 1.24c	218.5 ± 1.19c	9.3 ± 0.12ab	6.5 ± 0.05ab	9.1 ± 0.16b	72.8 (58.56)b	30.6 (33.58)a
Ptb33	R	38.2 ± 0.33a	71.1 ± 0.85a	3.8 ± 0.06a	2.9 ± 0.21a	4.5 ± 0.39a	26.6 (31.05)a	89.2 (70.81)cd
Swarna	HS	212.6 ± 1.20d	389.2 ± 1.39d	16.0 ± 0.17b	15.4 ± 0.78bc	14.9 ± 1.36c	96.1 (78.61)c	24.8 (29.87)a

**Notes:**

† Data in parenthesis are shown as Mean ± SE.

‡ The figures in parenthesis are transformed arcsine values.

R, Resistant; MR; Moderately resistant, MS: Moderately susceptible; HS, Highly susceptible.

The means indicated by different letters in a column are significantly different at *p* < 0.05 by Tukey’s HSD test.

#### Settling behaviour

The settling behaviour of BPH nymphs differed significantly among the tested landraces where the least number of nymphs settled on RL42, followed by Ptb33 and RL56 (Table 1). All most identical behaviour of nymphal settling was noticed on all the observation days. Overall, the number of nymphs settled 80.00% less on RL42, 78.12% on Ptb33 and 73.75% on RL4 concerning the susceptible check Swarna. The significantly lower number of adult males settled on RL42, Ptb33 and RL204, while Ptb33 and RL56 registered a significantly lower number of adult females of BPH (Table 1). The observations for both adult males and females were also found to be supplementary to the screening result of the landraces.

#### Nymphal survival

The mean percent survival rate of BPH nymphs on phenotypically resistant landraces was lower than on the Swarna (Table 1). The landraces such as RL4 (25.6%), RL35 (25.6%) and RL136 (27.1%) had the lowest survival rates, significantly different from Swarna (96.1%), but were statistically at par with Ptb33 (26.6%).

#### Hatching of eggs

Among the tested rice landraces, Ptb33 (89.2%), RL27 (83.4%), RL42 (81.2%) and RL4 (78.8%) exhibited significantly higher mean percent unhatched eggs of BPH, while Swarna registered the lowest percent (24.8%) of unhatched eggs and was not significantly different to RL118, RL162, RL109, RL209 and RL142 (Table 1).

### Biochemical components

#### Total phenol, reducing sugar and ascorbic acid

**Figure 2:**
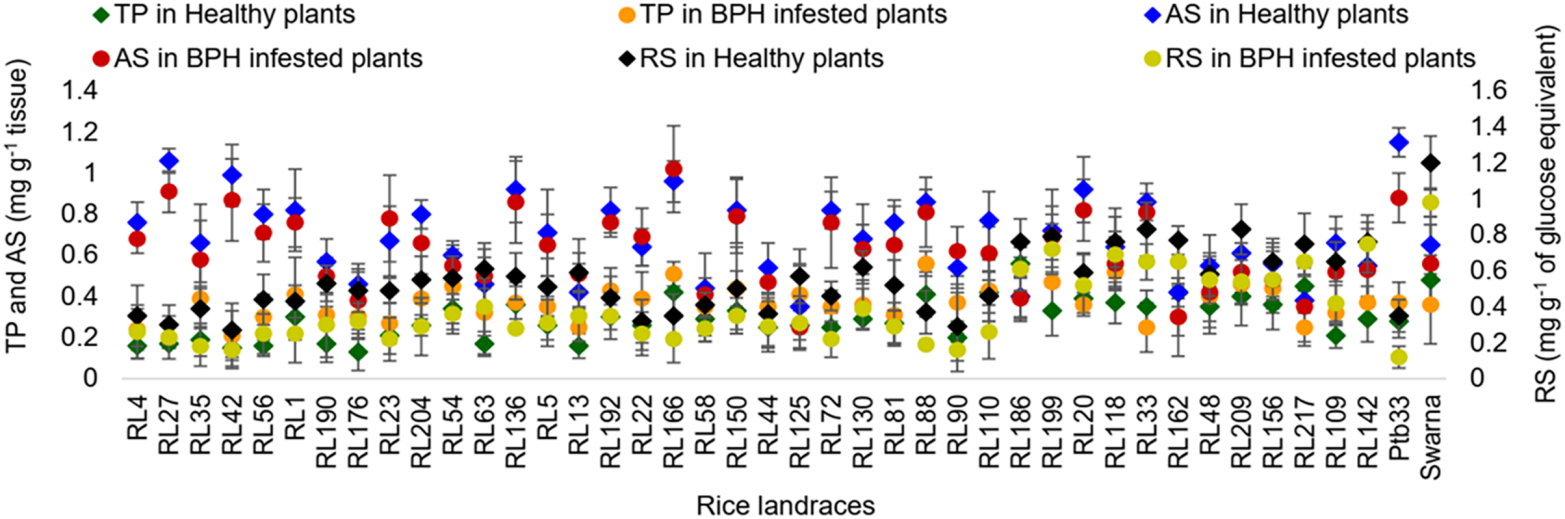
Relative variation in TP, RS and AS content before and 7 d after the BPH infestation in the selected rice landraces.

The total phenol (TP), reducing sugar (RS) and ascorbic acid (AS) content in the leaf sheaths of the BPH infested and healthy rice plants were estimated and differed significantly among the tested rice landraces (Fig. 2). In the healthy plants, TP content was found to be 0.28 mg g^−1^ tissue in Ptb33, whereas Swarna exhibited 0.48 mg g^−1^ tissue. After the BPH infestation, a percent increase in TP content varied significantly among the resistant rice landraces in the range between 15.01% and 51.28%. A significantly higher quantity of RS was observed in moderately susceptible landraces, with the highest in Swarna (1.20 mg g^−1^ glucose equivalent), compared to Ptb33 (0.35 mg g^−1^ glucose equivalent). After BPH feeding, a percent decrease in RS was observed in the range of 1.32% to 65.71%, irrespective of all the rice landraces including control varieties. Healthy leaf sheaths of Ptb33 (1.15 mg g^−1^ tissue) followed by RL27 (1.06 mg g^−1^ tissue) registered the significantly highest amount of AS content compared to Swarna (0.65 mg g^−1^ tissue), but the percent reduction of AS was observed to be 23.48%, 14.15% and 13.85% in Ptb33, RL27 and Swarna, respectively, after the BPH infestation.

#### Oxalic acid, crude silica and total free amino acid

**Figure 3:**
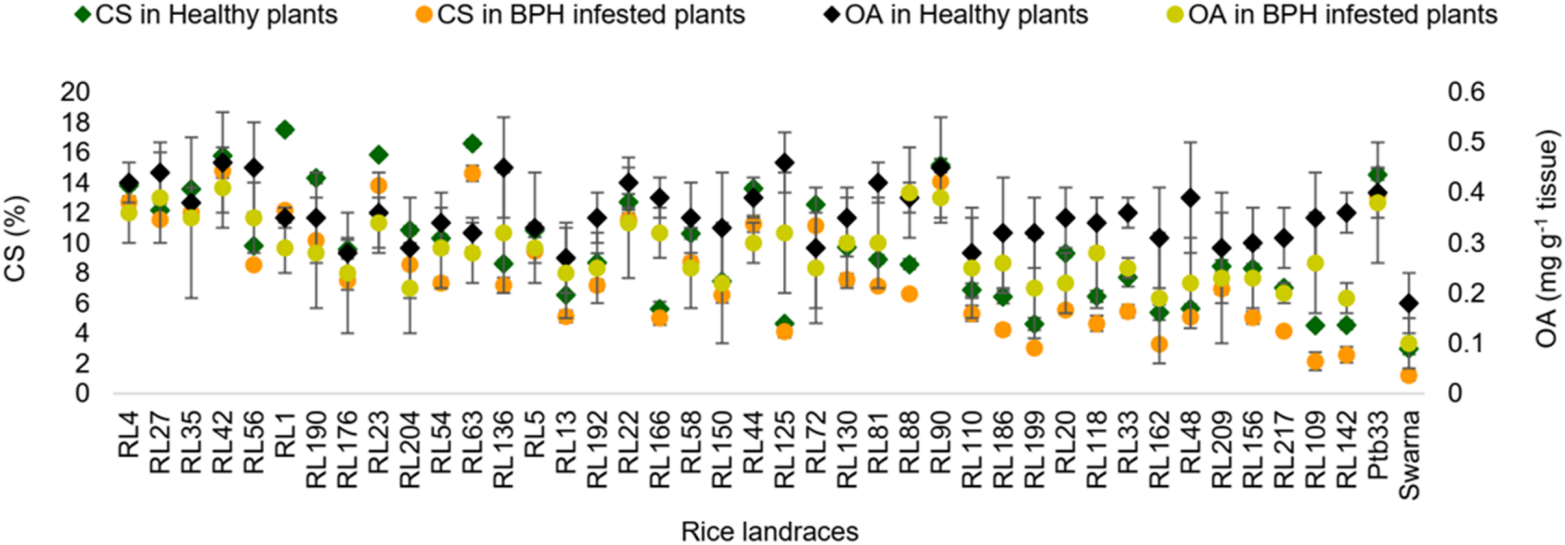
Relative variation in OA and CS content before and 7 d after the BPH infestation in the selected rice landraces.

**Figure 4:**
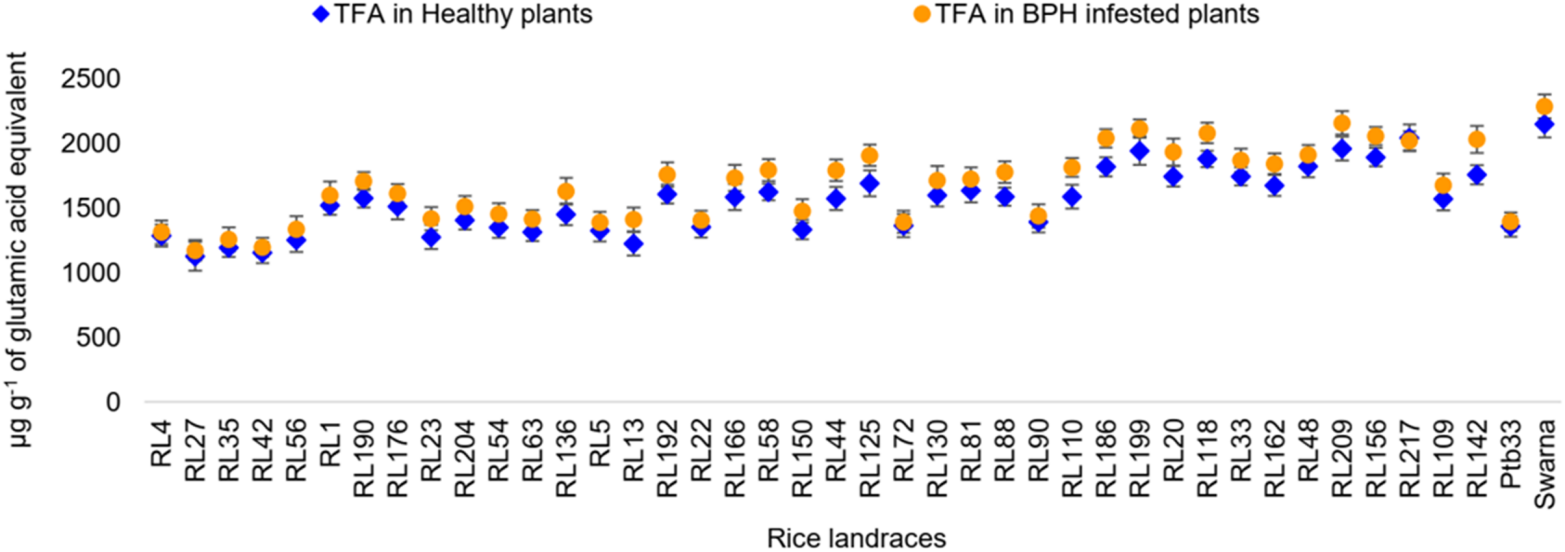
Relative variation in TFA content before and 7 d after the BPH infestation in the selected rice landraces.

In the healthy rice landraces, a significantly higher range of oxalic acid (OA) content was observed in resistant and moderately resistant rice landraces (0.27–0.46 mg g^−1^ tissue) compared to Swarna (0.18 mg g^−1^ tissue) (Fig. 3). Percent reduction in OA was observed irrespective of all the rice landraces, including the controls, after BPH feeding. Besides, RL1 (17.52%) and RL63 (16.60%) exhibited significantly higher crude silica (CS) content, found to be statistically at par with RL23 (15.85%), and RL42 (15.80%) and Ptb33 (14.53%), compared to Swarna. However, a significantly higher percent decrease in CS content was observed in moderately susceptible landraces than in resistant ones (Fig. 3). The significantly highest quantity of total free amino acid (TFA) was observed in Swarna (2,148.2 µg g^−1^ of glutamic acid equivalent) followed by RL217 (2,041.7 µg g^−1^ of glutamic acid equivalent), compared to the resistant landraces before BPH feeding (Fig. 4). However, infestation resulted in a significant increase in the quantity of TFA among the tested landraces, except RL217 (−1.14%).

#### Nitrogen, phosphorus and potassium

**Figure 5:**
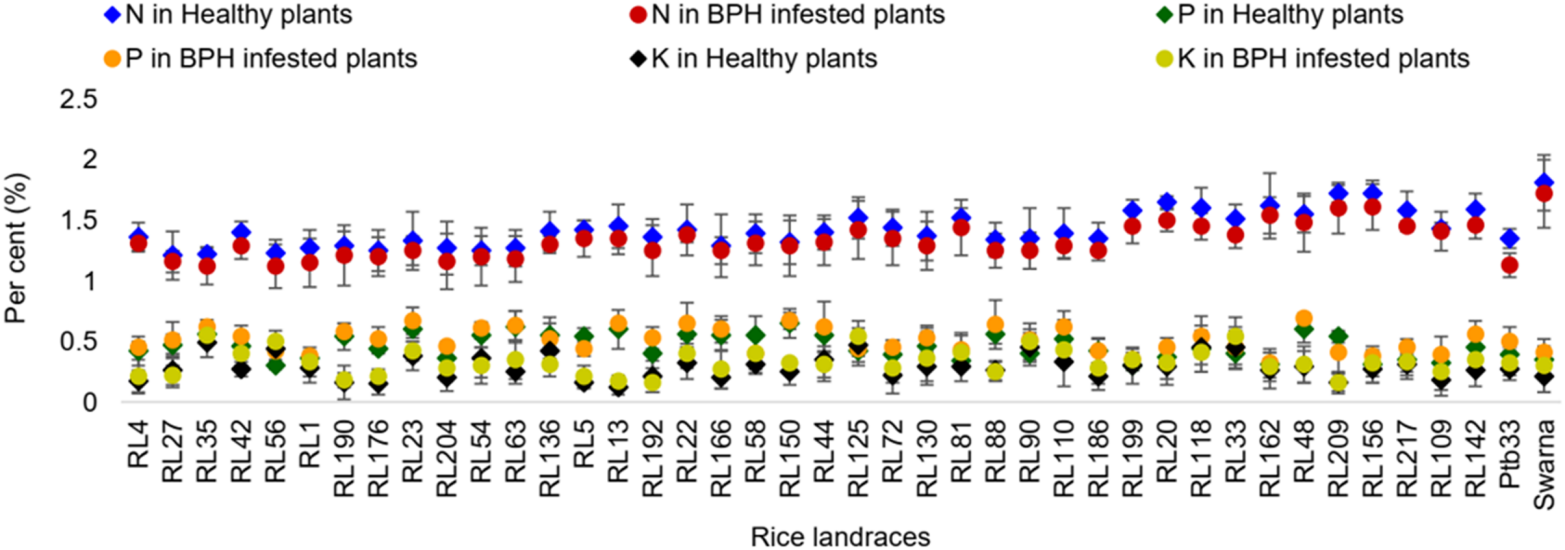
Relative variation in N, P and K content before and 7 d after the BPH infestation in the selected rice landraces.

The percent nitrogen (N) content was not varied significantly among the tested rice landraces, including controls, in healthy plants, while the BPH infested plants showed a significant variation (Fig. 5). Higher percent of N content was noticed in the moderately susceptible rice landraces (1.41% to 1.61%) with the highest in Swarna (1.72%), but the significantly lower range of N accumulation was observed in the resistant landraces (1.12% to 1.31%). However after BPH infestation, most of the resistant and moderately resistant landraces exhibited an increase in the percent phosphorus (P) content, except RL5 (−22.73%), and RL58 (−37.50%), RL209 (−31.71%) and RL136 (−5.77%). Additionally, a significant increase in potassium (K) content was observed in most of the rice landraces, however, RL209 exhibited a consistent rate in total K content before and after the BPH feeding (Fig. 5).

#### Correlation studies and principal component analysis

Pairwise correlation among the tested biochemical components quantified in various rice landraces indicated that the N was not significantly correlated with all the biochemical factors, except K (negatively) and TFA (positively) (Table 2). However, a significant and positive correlation was observed between TP and RS, K and OA, RS and TFA, AS and CS, and OA and CS. Additionally, N content in plants exhibited a significant and positive correlation with honeydew excretion and nymphal survival (Table 3). P was significantly but negatively correlated with nymphal survival, while percent un-hatched eggs, and settling of BPH nymphs and adult females showed a significantly positive and negative correlation, respectively, with both TP and K. Both OA and CS correlated significantly, but negatively, with honeydew excretion, settling of nymphs and adult females and nymphal survival. In contrast, honeydew excretion, settling of three BPH morphs and nymphal survival correlated significantly and positively with TFA (Table 3).

**Table 2:** Pairwise correlation coefficient comparison of the tested biochemical components.

	N	TP	P	K	RS	AS	OA	CS	TFA
N	1								
TP	0.11^ns^	1							
P	−0.34^ns^	0.12^ns^	1						
K	−0.06*	−0.01**	0.02^ns^	1					
RS	0.74^ns^	0.09*	−0.34^ns^	-0.06**	1				
AS	−0.34^ns^	0.04^ns^	0.18^ns^	−0.01^ns^	−0.40^ns^	1			
OA	−0.61^ns^	−0.10*	0.29^ns^	0.23*	−0.78*	0.45^ns^	1		
CS	−0.61^ns^	−0.32*	0.28^ns^	−0.11*	-0.73**	0.35**	0.69*	1	
TFA	0.72*	0.42^ns^	−0.34^ns^	−0.04**	0.79*	−0.41*	−0.67**	−0.76**	1

**Notes:**

* Significant at *p* < 0.05 level of significance.

** Significant at *p* < 0.01 level of significance.

ns, Non-significant; N, Nitrogen; TP, Total Phenol; P, Phosphorus; K, Potassium; RS, Reducing Sugar; AS, Ascorbic Acid; OA, Oxalic Acid; CS, Crude Silica; TFA, Total Free Amino Acid.

**Table 3:** Correlation between tested biochemical components and phenotypic reactions to BPH.

Parameters	N	TP	P	K	RS	AS	OA	CS	TFA
Excretion of honeydew	0.59**	−0.54*	−0.46^ns^	−0.72^ns^	0.66*	−0.52^ns^	−0.37**	−0.67*	0.61*
Nymphal settling	0.54^ns^	−0.76*	0.92^ns^	−0.56**	0.75*	-0.68^ns^	−0.72**	−0.59*	0.47*
Adult male settling	0.95^ns^	0.58*	−0.57^ns^	−0.37^ns^	0.69^ns^	−0.51*	−0.77*	−0.87^ns^	0.66*
Adult female settling	0.62^ns^	−0.64**	0.85^ns^	−0.48*	0.61**	−0.44^ns^	−0.54*	−0.68**	0.48**
Nymphal survival	0.81*	−0.68*	−0.67*	−0.52*	0.78*	−0.39^ns^	−0.97**	−0.81**	0.72*
Un-hatched eggs	−0.48^ns^	0.71*	0.44^ns^	0.78**	−0.42*	0.47**	0.80^ns^	0.73*	−0.55^ns^

**Notes:**

* Significant at *p* < 0.05 level of significance.

** Significant at *p* < 0.01 level of significance.

ns, Non-significant; N, Nitrogen; TP, Total Phenol; P, Phosphorus; K, Potassium; RS, Reducing Sugar; AS, Ascorbic Acid; OA, Oxalic Acid; CS, Crude Silica; TFA, Total Free Amino Acid.

**Figure 6:**
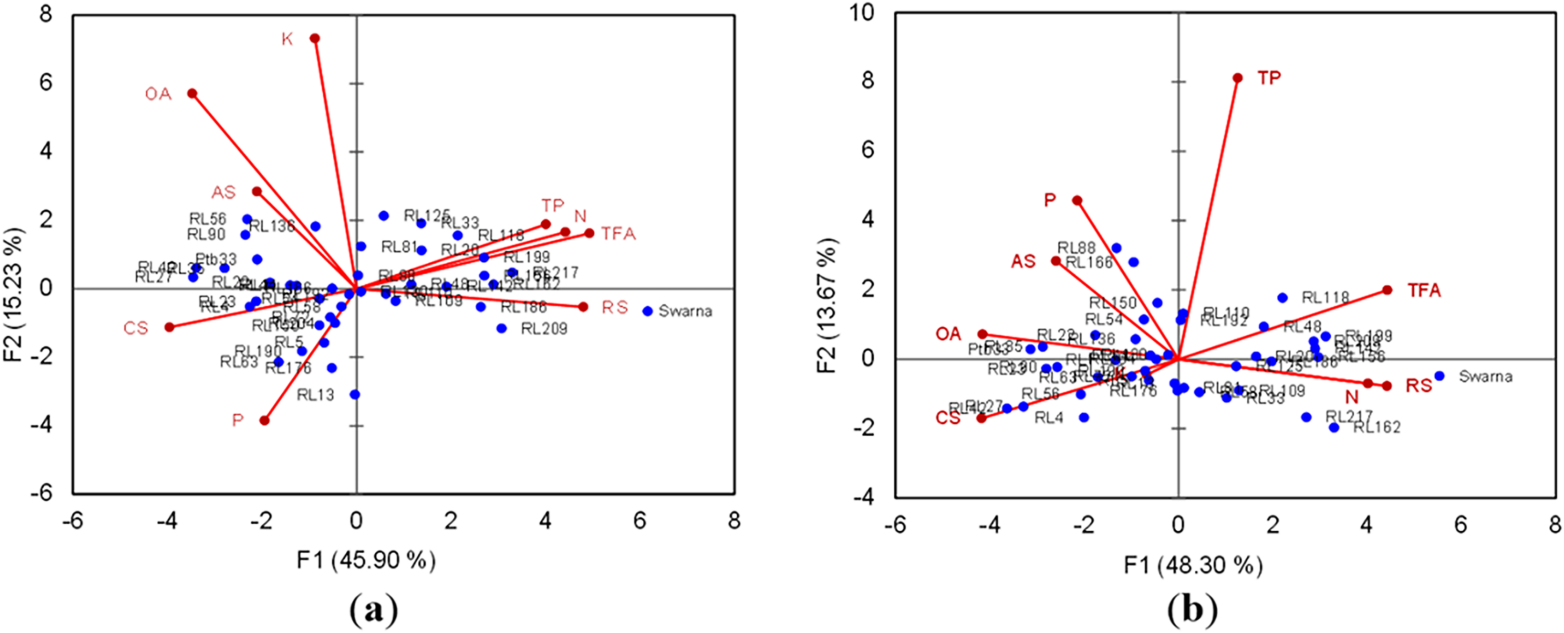
Scattered plot matrix score of healthy (A) and 7 d after BPH infested (B) rice landraces and tested biochemical components.

For healthy plants, it was clear from the biplot that resistant landraces were closely associated with P, CS, AS, OA and K, while TP, TFA, N and RS had a close association with Swarna (Fig. 6). In BPH infested plants, CS and OA exhibited their close intimacy with the resistant landraces, however, TP shifted away from the negative control variety. Total variance explained and principal factor matrix for tested feeding attributing biochemical components of BPH on rice landraces have been tabulated in (Tables S2 and S3).

## Discussion

The studies on rice resistance to BPH, one of the most notorious insect pests of rice in Asian countries, have been carrying out since long. Various traditional and wild rice varieties were identified as one of the major sources of resistant donors against BPH and reported by several workers through mass screening technique (Kalode & Khrishna, 1979; Jena et al., 2015). In the current study, the landraces with a score of ‘1’, neither preferred both nymphal and adult settling, encountered from the honeydew and settling tests, nor they were allowed for surviving and egg-laying (nymphal survivability and ovicidal test). These findings might be linked to the less ingestion of food and its improper usage impaired the development and survival of BPH on resistant varieties (Alagar et al., 2007). Feeding can only be determined preciously through computing the area of honeydew excretion and several studies recognized this method as the best for complementing the phenotypic screening (He et al., 2013). Various plant metabolites present within resistant rice cultivars inhibit the feeding activities of BPH due to the less preference and that was reflected in low honeydew excretion (He et al., 2013). Besides resistant cultivars, significantly lower amounts of honeydew excreted by BPH, when feeding on moderately resistant landraces, confirmed the accuracy of phenotypic screening (Singh, Sarao & Sharma, 2017) and the possible trend of resistance among the respective rice landraces (Ritu & Ravi, 2006). Soundarajan, Gunathilagaraj & Chitra (2002) also conceded that the enumeration of the feeding rate of BPH is a potential indicator to differentiate the resistant and susceptible genotypes of rice.

Results of the present study showed that a comparatively lower percent nymphal population of BPH survived on phenotypically resistant rice landraces than Swarna. These results were corroborated by the findings of Vanitha, Suresh & Gunathilagaraj (2011) and Kumar, Maurya & Tiwari (2012), who confirmed the reduced survival and longevity of BPH nymphs and adults on resistant and moderately resistant genotypes. Reduced and poor survival of BPH might be due to the lower feeding rate on resistant landraces, which may be attributable to the lack of phagostimulant or presence of antifeedant components (Seo et al., 2009; He et al., 2013; Sable, Suresh & Kumar, 2015). However, there could also be a great possibility of the inadequacy of essential plant nutrients in these rice landraces, required for BPH survival. In addition, several physiochemical factors of resistant landraces play an important role in insect herbivory by preventing ingestion of the adequate foods and nutrients required for insect survival (Bing et al., 2007; Syobu, Otuka & Matsumura, 2011). On the other hand, a significantly lower survival of BPH nymphs in a rice variety SD15 might be linked to the higher rate of unhatched eggs, and thus tends to be resistant to the diverse adaptabilities of the host (Zheng et al., 2017). However, antibiotic resistance levels in some resistant rice accessions were positively associated with the quantity of BPH feeding (Hao et al., 2008; Yang et al., 2017; Han et al., 2018). Therefore, different resistant factors among various selected rice landraces could be associated with the antibiotic reactions against BPH (Darshini & Sidde Gowda, 2015).

Nitrogen (N) content is regarded as an indicator of plant quality which was reported to induce a barrier against the resistance of BPH in rice (Salim, 2002; Lu & Heong, 2009). The higher quantity of honeydew excretion by BPH was obtained in susceptible cultivars, and N was significant and positively correlated with this behaviour. The synergistic relationship between N in rice leaf and higher feeding rate of BPH is possibly due to the ready-made succulence in leaf sheath for higher N content which may not affect the insect biology directly, but changes the host biochemistry and plays a significant role in the reduction of plant resistance (Rashid, Jahan & Islam, 2016). Results of the present study on the role and impact of N against BPH are also consistent with the elaborative findings of Lu et al. (2004, 2005) and Horgan et al. (2018). Besides N, phosphorus (P) and potassium (K) are also required by the herbivores for ATP and nucleic acid synthesis along with several physiological activities. A significant and negative impact of K on BPH feeding, settling, survival and reproduction observed in the present study, may be attributable to the distribution of primary metabolites in plant tissues, which in turn could affect the attractiveness of the plant for insects as well as their subsequent growth and development on it (Rashid et al., 2017a). However, some workers confirmed that a higher level of K was associated with a lower population of BPH due to the reduced level of reducing sugar (RS) and total free amino acid (TFA) in K rich rice cultivars (Vanitha, Suresh & Gunathilagaraj, 2011), and strongly boosted the correlation and principal component analysis (PCA) of the present investigation. The phenolic compounds were found to be the feeding deterrents to BPH in rice and generally have a positive correlation with host plant resistance (Singh, 2004). In the present study, a significantly lower level of total phenol (TP) was observed in the resistant landraces, compared to the susceptible check, where a higher percent increase in TP took place in the BPH infested resistant landraces. The implication of phloem chemistry of rice comprises silicic acid, oxalic acid and phenolic compounds, provokes resistance to BPH (Ghaffar, Pritchard & Ford-Lloyd, 2011), but the latter usually possess a negative impact over the formers (Ciulu, Cadiz-Gurrea & Segura-Carretero, 2018). Grayer et al. (1994) reported that higher silicon content in rice leaf sheath of a resistant variety can reduce the TP content at a lower level without disrupting the phenotypic resistance of the concerned rice variety to BPH. In addition, both oxalic acid (OA) and crude silica (CS) were already recognized as the sucking inhibitor against BPH in rice, and in the present experiment, a significant and positive correlation was observed between them. For BPH, reduced performance with impaired feeding behaviours and poor population growth on rice was recorded in higher silicon content cultivars by some previous workers (He et al., 2015; Reynolds et al., 2016; Yang et al., 2017) and strongly supported our findings. The possible mechanisms of plant resistance related to higher silicon content may be the increased rigidity and reduced digestibility of plant tissues due to a physical barrier formed from the higher deposition of silica in epidermal cells of resistant rice plants (Massey, Ennos & Hartley, 2006; Massey & Hartley, 2009; Han et al., 2015). Moreover, this physical barrier has the potential to reduce the food quality of herbivores and thus impair their feeding capability followed by the reduction of insect growth rate (Calandra et al., 2016). The TFA also played a significant role in BPH infestation on rice where most of the resistant landraces, including Ptb33, registered lower TFA content. It may be attributed that, resistant cultivars against sap suckers usually possess a lower quantity of TFA by limiting the nutritive value of plant tissues for the herbivores (Golan et al., 2017). Biplot of PCA suggests that TFA, RS and N content were in a close association in the healthy rice plants, while the distance between the former and two later was largest after BPH feeding. It was evident that the level of TFA content in rice leaf sheath increased after the BPH infestation which is consistent with the findings of Sempruch, Michalak & Leszczynski (2011). Although it is still not clear by the researchers regarding the mechanisms of resource allocation when attacked by herbivores, it can be hypothesized that higher cell damage would make the plant resource sequestration a possible preferred strategy (Orians, Thorn & Gomez, 2011). Moreover, Rashid et al. (2017b) linked higher K content with a lower level of TFA in the resistant rice plants and observed the increment of both the compounds after BPH feeding.

## Conclusions

In conclusion, a high level of plant defence against BPH biotype 4, through antibiosis, antixenosis and biochemical bases of resistance, was exhibited by five phenotypically resistant rice landraces (RL4, RL27, RL35, RL42 and RL56), which may provide a useful tool for the plant breeders and biotechnologists to develop BPH resistant rice varieties. Besides, the restless behaviour of BPH on the phenotypically resistant rice landraces could enhance the chance of predation by the natural enemies in the field. Understanding the phenotypic expressions and biochemical mechanisms underlying resistance in rice landraces will contribute to the effective management of BPH biotype 4 and facilitate resistance breeding program more efficiently in future.

## Supplemental Information

10.7717/peerj.14360/supp-1 Supplemental Information 1 Supplemental Tables and Raw Data. Raw data generated during the three years screening of 218 rice landraces in both greenhouse and open-field conditions, used to develop the cluster dendrogram.

## Additional Information and Declarations

### Data Availability

The following information was supplied regarding data availability: The raw data are available in the Supplemental Files.
