# Supplementary material for: Evaluation and characterization of indigenous rice (Oryza sativa L.) landraces resistant to brown planthopper Nilaparvata lugens (Stål.) biotype 4
Source: PeerJ. 2022 Nov 4;10:e14360. doi: 10.7717/peerj.14360 (PMC9639428; doi:10.7717/peerj.14360)
Supplement: Supplemental Information 1 — Raw data generated during the three years screening of 218 rice landraces in both greenhouse and open-field conditions, used to develop the cluster dendrogram. [file peerj-10-14360-s001.docx]

**Supplemental Tables**

**Table S1:** Raw data of the three years screening of selected rice landraces

| **Type** | **Designation*** | **Damage score** | | | | | | | | | **No. of BPH/ 3 plants (mean)** | **% chaffy grain (mean)** | **Location (District, State)** |
| --- | --- | --- | --- | --- | --- | --- | --- | --- | --- | --- | --- | --- | --- |
|  |  | **Free-choice screening** | | | **No-choice screening** | | | **Field screening** | | |  |  |  |
|  |  | **2017** | **2018** | **2019** | **2017** | **2018** | **2019** | **2017** | **2018** | **2019** |  |  |  |
| RL1 | Adanshilpa/FRV/8-223 | 3.1^MR^ | 3.3 ^MR^ | 3.4 ^MR^ | 3.8 ^MR^ | 4.2 ^MR^ | 3.2 ^MR^ | 3.2 ^MR^ | 3.6 ^MR^ | 3.5 ^MR^ | 9.2 | 5.2 | Bankura, WB |
| RL2 | Agniban/FRV/8-225 | 4.2 ^MR^ | 4.6 ^MR^ | 3.9 ^MR^ | 4.6 ^MR^ | 5.3 ^MS^ | 5.1 ^MS^ | 3.9 ^MR^ | 4.0 ^MR^ | 3.1 ^MR^ | 12.4 | 6.5 | Nadia, WB |
| RL180 | Agnishal/FRV/8-273 | 4.1 ^MR^ | 4.2 ^MR^ | 4.7 ^MR^ | 4.7 ^MR^ | 4.5 ^MR^ | 6.2 ^MS^ | 3.1 ^MR^ | 3.9 ^MR^ | 3.8 ^MR^ | 12.3 | 5.2 | Purulia, WB |
| RL3 | Anshfal/FRV/9-281 | 5.3 ^MS^ | 6.9 ^MS^ | 6.3 ^MS^ | 6.2 ^MS^ | 6.9 ^MS^ | 7.3^S^ | 5.2 ^MS^ | 5.4 ^MS^ | 6.0 ^MS^ | 20.3 | 12.5 | Purulia, WB |
| RL181 | Anshkata/FRV/10-355 | 3.8 ^MR^ | 3.6 ^MR^ | 4.6 ^MR^ | 4.5 ^MR^ | 5.1 ^MS^ | 5.3 ^MS^ | 3.5 ^MR^ | 3.8 ^MR^ | 3.7 ^MR^ | 16.5 | 7.0 | Bankura, WB |
| RL182 | Ashu/FRV/6-160 | 9.0 ^HS^ | 9.0 ^HS^ | 9.0 ^HS^ | 8.3 ^S^ | 8.1 ^S^ | 9.0 ^HS^ | 9.0 ^HS^ | 9.0 ^HS^ | 9.0 ^HS^ | 34.1 | 24.0 | West Midnapore, WB |
| RL183 | Ashwinjharia/FRV/6-161 | 6.2 ^MS^ | 5.5 ^MS^ | 6.0 ^MS^ | 6.6 ^MS^ | 7.9 ^S^ | 7.1 ^S^ | 6.8 ^MS^ | 6.3 ^MS^ | 5.8 ^MS^ | 25.8 | 14.9 | Bankura, WB |
| RL184 | Ashwinshal/FRV/7-214 | 7.8 ^S^ | 8.0 ^S^ | 7.9 ^S^ | 7.2 ^S^ | 6.3 ^MS^ | 9.0 ^HS^ | 7.9 ^S^ | 8.1 ^S^ | 8.6 ^S^ | 29.0 | 19.8 | Bankura, WB |
| RL155 | Bachi/FRV/7-213 | 5.2 ^MS^ | 6.1 ^MS^ | 6.7 ^MS^ | 8.2 ^S^ | 7.0 ^S^ | 6.1 ^MS^ | 6.3 ^MS^ | 6.9 ^MS^ | 6.1 ^MS^ | 17.1 | 13.6 | Purulia, WB |
| RL113 | Badamsaru/FRV/9-306 | 5.7 ^MS^ | 7.7 ^S^ | 7.6 ^S^ | 6.1 ^MS^ | 6.6 ^MS^ | 7.8 ^S^ | 4.5 ^MR^ | 5.0 ^MS^ | 4.9 ^MR^ | 19.3 | 6.9 | Bankura, WB |
| RL185 | Badkalam/FRV/8-274 | 4.2 ^MR^ | 3.7 ^MR^ | 5.0 ^MS^ | 3.9 ^MR^ | 3.6 ^MR^ | 5.7 ^MS^ | 3.6 ^MR^ | 4.6 ^MR^ | 4.2 ^MR^ | 19.3 | 8.6 | Purulia, WB |
| RL186 | Badkalamkathi/FRV/9-320 | 5.1 ^MS^ | 5.9 ^MS^ | 5.2 ^MS^ | 3.6 ^MR^ | 3.9 ^MR^ | 4.2 ^MR^ | 4.0 ^MR^ | 4.5 ^MR^ | 4.8 ^MR^ | 13.9 | 7.6 | Birbhum, WB |
| RL4 | Badshabhog/FRV/8-226 | 2.2 ^R^ | 2.7 ^R^ | 1.8 ^R^ | 2.8 ^R^ | 3.2 ^MR^ | 3.9 ^MR^ | 1.9 ^R^ | 2.0 ^R^ | 2.1 ^R^ | 10.2 | 3.6 | South 24 Pgs, WB |
| RL5 | Bahurupi/FRV/7-171 | 3.3 ^MR^ | 3.8 ^MR^ | 4.0 ^MR^ | 4.2 ^MR^ | 4.9 ^MR^ | 4.0 ^MR^ | 3.3 ^MR^ | 3.0 ^MR^ | 3.7 ^MR^ | 7.3 | 5.1 | Bankura, WB |
| RL6 | Balam/FRV/6-107 | 5.1 ^MS^ | 6.0 ^MS^ | 6.2 ^MS^ | 6.8 ^MS^ | 5.9 ^MS^ | 6.1 ^MS^ | 5.3 ^MS^ | 5.0 ^MS^ | 5.4 ^MS^ | 18.2 | 10.7 | Birbhum, WB |
| RL187 | Balaramshal/FRV/9-322 | 3.2 ^MR^ | 5.1 ^MS^ | 4.5 ^MR^ | 5.0 ^MS^ | 4.8 ^MR^ | 6.1 ^MS^ | 4.0 ^MR^ | 4.0 ^MR^ | 4.3 ^MR^ | 14.9 | 6.9 | Bankura, WB |
| RL156 | Banshkamini/FRV/6-146 | 6.2 ^MS^ | 5.1 ^MS^ | 5.3 ^MS^ | 5.1 ^MS^ | 5.2 ^MS^ | 4.9 ^MR^ | 4.6 ^MR^ | 4.2 ^MR^ | 4.4 ^MR^ | 15.8 | 9.3 | Purulia, WB |
| RL7 | Banshkathi/FRV/8-228 | 6.1 ^MS^ | 6.6 ^MS^ | 5.6 ^MS^ | 7.0 ^S^ | 6.2 ^MS^ | 6.8 ^MS^ | 5.5 ^MS^ | 6.1 ^MS^ | 6.3 ^MS^ | 22.6 | 14.3 | Bankura, WB |
| RL9 | Banshpata/FRV/7-174 | 7.9 ^S^ | 8.2 ^S^ | 8.6 ^S^ | 8.2 ^S^ | 7.9 ^S^ | 8.5 ^S^ | 7.2 ^S^ | 7.6 ^S^ | 7.6 ^S^ | 30.6 | 20.2 | Purulia, WB |
| RL8 | Banshphul/FRV/9-283 | 5.7 ^MS^ | 7.0 ^S^ | 6.0 ^MS^ | 5.7 ^MS^ | 6.4 ^MS^ | 6.3 ^MS^ | 6.2 ^MS^ | 5.7 ^MS^ | 5.9 ^MS^ | 16.8 | 12.1 | Bankura, WB |
| RL188 | Barajashoa/FRV/4-047 | 9.0 ^HS^ | 9.0 ^HS^ | 9.0 ^HS^ | 9.0 ^HS^ | 9.0 ^HS^ | 9.0 ^HS^ | 9.0 ^HS^ | 9.0 ^HS^ | 9.0 ^HS^ | 32.7 | 24.6 | Bankura, WB |
| RL158 | Barani/FRV/6-149 | 9.0 ^HS^ | 9.0 ^HS^ | 9.0 ^HS^ | 9.0 ^HS^ | 9.0 ^HS^ | 7.8 ^S^ | 9.0 ^HS^ | 9.0 ^HS^ | 9.0 ^HS^ | 36.2 | 24.1 | Purulia, WB |
| RL10 | Basamanik/FRV/5-056 | 4.8 ^MR^ | 4.0 ^MR^ | 3.9 ^MR^ | 4.6 ^MR^ | 5.8 ^MS^ | 5.1 ^MS^ | 4.1 ^MR^ | 3.9 ^MR^ | 4.6 ^MR^ | 15.5 | 7.5 | West Midnapore, WB |
| RL11 | Bekur/FRV/10-328 | 8.8 ^S^ | 7.9 ^S^ | 8.0 ^S^ | 7.6 ^S^ | 8.6 ^S^ | 8.2 ^S^ | 8.0 ^S^ | 7.6 ^S^ | 7.9 ^S^ | 24.5 | 17.3 | South 24 Pgs, WB |
| RL157 | Bhadoi/FRV/6-148 | 4.6 ^MR^ | 3.5 ^MR^ | 3.9 ^MR^ | 4.2 ^MR^ | 3.8 ^MR^ | 5.6 ^MS^ | 3.6 ^MR^ | 3.9 ^MR^ | 3.5 ^MR^ | 12.6 | 4.9 | Bankura, WB |
| RL12 | Bhalki/FRV/8-230 | 3.2 ^MR^ | 4.9 ^MR^ | 4.5 ^MR^ | 5.3 ^MS^ | 6.1 ^MS^ | 4.9 ^MR^ | 3.9 ^MR^ | 4.0 ^MR^ | 3.7 ^MR^ | 16.7 | 8.3 | Bankura, WB |
| RL114 | Bhurishal/FRV/11-366 | 8.6 ^S^ | 8.2 ^S^ | 8.5 ^S^ | 9.0 ^HS^ | 7.9 ^S^ | 8.5 ^S^ | 8.2 ^S^ | 7.7 ^S^ | 8.0 ^S^ | 26.1 | 19.8 | Purulia, WB |
| RL189 | Bhutia/FRV/9-323 | 4.2 ^MR^ | 3.9 ^MR^ | 4.8 ^MR^ | 4.5 ^MR^ | 5.4 ^MS^ | 5.6 ^MS^ | 3.9 ^MR^ | 4.2 ^MR^ | 4.3 ^MR^ | 15.3 | 7.7 | West Midnapore, WB |
| RL13 | Bhutmuri/FRV/14-393 | 4.0 ^MR^ | 3.1 ^MR^ | 4.6 ^MR^ | 4.2 ^MR^ | 3.8 ^MR^ | 4.8 ^MR^ | 4.5 ^MR^ | 4.0 ^MR^ | 3.1 ^MR^ | 10.5 | 5.5 | West Midnapore, WB |
| RL190 | Binni/FRV/9-324 | 3.7 ^MR^ | 4.0 ^MR^ | 4.6 ^MR^ | 6.1 ^MS^ | 3.7 ^MR^ | 4.9 ^MR^ | 4.0 ^MR^ | 3.6 ^MR^ | 4.1 ^MR^ | 16.2 | 6.1 | Coochbehar, WB |
| RL14 | Birohi/FRV/12-370 | 5.9 ^MS^ | 6.2 ^MS^ | 6.4 ^MS^ | 6.4 ^MS^ | 5.8 ^MS^ | 5.6 ^MS^ | 5.9 ^MS^ | 6.2 ^MS^ | 6.0 ^MS^ | 20.9 | 11.2 | Birbhum, WB |
| RL15 | Birol/FRV/6-109 | 3.6 ^MR^ | 4.8 ^MR^ | 4.1 ^MR^ | 5.3 ^MS^ | 6.2 ^MS^ | 6.5 ^MS^ | 3.2 ^MR^ | 3.9 ^MR^ | 3.5 ^MR^ | 20.1 | 6.2 | Bankura, WB |
| RL191 | Botadhan/FRV/8-276 | 4.8 ^MR^ | 4.6 ^MR^ | 3.9 ^MR^ | 5.8 ^MS^ | 4.2 ^MR^ | 4.9 ^MR^ | 3.3 ^MR^ | 3.9 ^MR^ | 3.7 ^MR^ | 11.9 | 6.3 | Coochbehar, WB |
| RL192 | Boubhog/FRV/9-325 | 4.2 ^MR^ | 3.3 ^MR^ | 4.5 ^MR^ | 5.6 ^MS^ | 6.1 ^MS^ | 6.7 ^MS^ | 8.2 ^S^ | 8.1 ^S^ | 7.8 ^S^ | 19.1 | 21.0 | Purulia, WB |
| RL193 | Burikankri/FRV/10-356 | 5.6 ^MS^ | 3.1 ^MR^ | 3.9 ^MR^ | 4.6 ^MR^ | 5.7 ^MS^ | 6.3 ^MS^ | 4.8 ^MR^ | 3.1 ^MR^ | 3.0 ^MR^ | 16.7 | 7.5 | West Midnapore, WB |
| RL194 | Chaitannya/FRV/4-049 | 7.6 ^S^ | 7.6 ^S^ | 7.2 ^S^ | 7.1 ^S^ | 5.9 ^MS^ | 6.2 ^MS^ | 5.0 ^MS^ | 5.0 ^MS^ | 4.1 ^MR^ | 20.4 | 7.9 | Bankura, WB |
| RL164 | Chakramala/FRV/9-314 | 3.8 ^MR^ | 4.6 ^MR^ | 4.9 ^MR^ | 4.9 ^MR^ | 5.2 ^MS^ | 5.6 ^MS^ | 4.3 ^MR^ | 4.0 ^MR^ | 3.0 ^MR^ | 14.8 | 7.2 | Birbhum, WB |
| RL197 | Chamak/FRV/5-101 | 7.6 ^S^ | 8.8 ^S^ | 8.5 ^S^ | 5.1 ^MS^ | 8.7 ^S^ | 9.0 ^HS^ | 7.5 ^S^ | 8.6 ^S^ | 8.2 ^S^ | 25.2 | 19.2 | Nadia, WB |
| RL16 | Chamanmuni/FRV/9-284 | 7.2 ^S^ | 8.6 ^S^ | 8.5 ^S^ | 9.0 ^HS^ | 8.5 ^S^ | 8.9 ^S^ | 7.2 ^S^ | 8.6 ^S^ | 8.5 ^S^ | 26.1 | 17.9 | East Midnapore, WB |
| RL17 | Chamatkar/FRV/9-290 | 6.0 ^MS^ | 5.2 ^MS^ | 6.7 ^MS^ | 7.2 ^S^ | 6.7 ^MS^ | 7.0 ^S^ | 6.6 ^MS^ | 6.9 ^MS^ | 6.0 ^MS^ | 18.2 | 11.7 | South 24 Pgs, WB |
| RL18 | Chandrakanta/FRV/10-330 | 6.2 ^MS^ | 6.8 ^MS^ | 6.9 ^MS^ | 6.1 ^MS^ | 5.9 ^MS^ | 6.6 ^MS^ | 5.6 ^MS^ | 5.5 ^MS^ | 6.2 ^MS^ | 13.3 | 10.1 | Bankura, WB |
| RL195 | Changarangi/FRV/4-050 | 3.4 ^MR^ | 4.6 ^MR^ | 4.9 ^MR^ | 4.6 ^MR^ | 5.6 ^MS^ | 4.3 ^MR^ | 3.6 ^MR^ | 3.9 ^MR^ | 4.2 ^MR^ | 13.1 | 5.7 | South 24 Pgs, WB |
| RL196 | Chapakushi/FRV/4-052 | 9.0 ^HS^ | 9.0 ^HS^ | 9.0 ^HS^ | 9.0 ^HS^ | 9.0 ^HS^ | 9.0 ^HS^ | 8.8 ^S^ | 9.0 ^HS^ | 9.0 ^HS^ | 35.9 | 26.0 | East Midnapore, WB |
| RL116 | Chhotodidi/FRV/10-342 | 6.2 ^MS^ | 6.0 ^MS^ | 5.6 ^MS^ | 5.6 ^MS^ | 5.8 ^MS^ | 7.4 ^S^ | 5.8 ^MS^ | 6.3 ^MS^ | 6.5 ^MS^ | 16.8 | 12.8 | Purulia, WB |
| RL176 | Chikonmashuri/FRV/5-096 | 3.2 ^MR^ | 4.0 ^MR^ | 4.7 ^MR^ | 3.6 ^MR^ | 5.8 ^MS^ | 5.5 ^MS^ | 3.1 ^MR^ | 3.2 ^MR^ | 4.0 ^MR^ | 10.5 | 6.8 | Purulia, WB |
| RL115 | Chinakamini/FRV/11-367 | 7.4 ^S^ | 7.9 ^S^ | 8.2 ^S^ | 8.6 ^S^ | 8.4 ^S^ | 8.0 ^S^ | 8.1 ^S^ | 7.9 ^S^ | 7.5 ^S^ | 29.5 | 21.0 | South 24 Pgs, WB |
| RL143 | Chiniatap/FRV/7-210 | 7.9 ^S^ | 8.0 ^S^ | 7.6 ^S^ | 8.1 ^S^ | 8.5 ^S^ | 8.7 ^S^ | 7.9 ^S^ | 8.2 ^S^ | 8.6 ^S^ | 26.1 | 19.5 | Purulia, WB |
| RL144 | Chinishankar/FRV/8-265 | 8.1 ^S^ | 7.4 ^S^ | 8.5 ^S^ | 8.3 ^S^ | 7.0 ^S^ | 8.9 ^S^ | 8.2 ^S^ | 7.6 ^S^ | 7.0 ^S^ | 24.5 | 18.6 | Purulia, WB |
| RL177 | Cholish/FRV/5-099 | 7.7 ^S^ | 8.2 ^S^ | 8.0 ^S^ | 9.0 ^HS^ | 8.1 ^S^ | 6.3 ^MS^ | 8.6 ^S^ | 7.2 ^S^ | 8.1 ^S^ | 28.9 | 18.4 | South 24 Pgs, WB |
| RL198 | Churnakathi/FRV/5-102 | 4.6 ^MR^ | 4.2 ^MR^ | 4.1 ^MR^ | 5.2 ^MS^ | 6.8 ^MS^ | 5.5 ^MS^ | 4.6 ^MR^ | 4.0 ^MR^ | 4.4 ^MR^ | 19.3 | 8.9 | Bankura, WB |
| RL117 | Danaguri/FRV/9-307 | 3.6 ^MR^ | 4.5 ^MR^ | 4.9 ^MR^ | 5.3 ^MS^ | 5.9 ^MS^ | 4.8 ^MR^ | 3.6 ^MR^ | 4.8 ^MR^ | 4.1 ^MR^ | 15.8 | 8.4 | Bankura, WB |
| RL19 | Datshal/FRV/8-232 | 6.1 ^MS^ | 6.5 ^MS^ | 6.7 ^MS^ | 5.2 ^MS^ | 6.7 ^MS^ | 6.5 ^MS^ | 6.5 ^MS^ | 5.9 ^MS^ | 6.1 ^MS^ | 17.2 | 13.2 | South 24 Pgs, WB |
| RL199 | Dayalmadina/FRV/6-162 | 6.1 ^MS^ | 6.2 ^MS^ | 4.3 ^MR^ | 4.5 ^MR^ | 5.1 ^MS^ | 4.5 ^MR^ | 4.2 ^MR^ | 3.8 ^MR^ | 4.9 ^MR^ | 11.5 | 7.2 | Birbhum, WB |
| RL20 | Deradun gandheshwari/FRV/11-357 | 4.1 ^MR^ | 4.3 ^MR^ | 5.4 ^MS^ | 4.2 ^MR^ | 4.7 ^MR^ | 5.1 ^MS^ | 4.2 ^MR^ | 4.8 ^MR^ | 4.9 ^MR^ | 10.7 | 7.6 | Bankura, WB |
| RL159 | Dharanshal/FRV/4-043 | 3.7 ^MR^ | 4.2 ^MR^ | 4.6 ^MR^ | 5.2 ^MS^ | 5.7 ^MS^ | 6.1 ^MS^ | 4.7 ^MR^ | 4.0 ^MR^ | 4.1 ^MR^ | 18.3 | 6.4 | Bankura, WB |
| RL21 | Dorangi/FRV/7-175 | 4.0 ^MR^ | 3.8 ^MR^ | 4.4 ^MR^ | 5.7 ^MS^ | 6.8 ^MS^ | 4.5 ^MR^ | 4.0 ^MR^ | 3.5 ^MR^ | 4.2 ^MR^ | 17.2 | 6.9 | South 24 Pgs, WB |
| RL22 | Dudheshwar/FRV/11-358 | 3.9 ^MR^ | 4.2 ^MR^ | 4.8 ^MR^ | 4.2 ^MR^ | 3.9 ^MR^ | 5.8 ^MS^ | 7.3 ^S^ | 8.0 ^S^ | 7.7 ^S^ | 8.2 | 15.8 | South 24 Pgs, WB |
| RL118 | Dudhkalam/FRV/8-259 | 4.7 ^MR^ | 4.8 ^MR^ | 4.0 ^MR^ | 5.1 ^MS^ | 5.0 ^MS^ | 4.7 ^MR^ | 4.5 ^MR^ | 4.1 ^MR^ | 4.8 ^MR^ | 16.6 | 9.7 | Bankura, WB |
| RL200 | Dular/FRV/7-216 | 8.5 ^S^ | 8.4 ^S^ | 8.0 ^S^ | 6.3 ^MS^ | 8.7 ^S^ | 8.8 ^S^ | 8.8 ^S^ | 8.2 ^S^ | 8.5 ^S^ | 28.8 | 20.5 | Hooghly, WB |
| RL201 | Dumurkandi/FRV/4-054 | 3.8 ^MR^ | 4.7 ^MR^ | 4.0 ^MR^ | 4.6 ^MR^ | 5.0 ^MS^ | 4.2 ^MR^ | 3.8 ^MR^ | 3.9 ^MR^ | 4.0 ^MR^ | 13.2 | 7.6 | Birbhum, WB |
| RL166 | Dumurshal/FRV/6-152 | 3.7 ^MR^ | 3.9 ^MR^ | 4.9 ^MR^ | 6.1 ^MS^ | 5.1 ^MS^ | 5.8 ^MS^ | 7.4 ^S^ | 7.7 ^S^ | 7.5 ^S^ | 15.3 | 19.1 | Purulia, WB |
| RL146 | Gaganshal/FRV/8-266 | 4.2 ^MR^ | 3.3 ^MR^ | 3.8 ^MR^ | 5.6 ^MS^ | 6.8 ^MS^ | 5.7 ^MS^ | 4.6 ^MR^ | 4.8 ^MR^ | 4.0 ^MR^ | 10.2 | 5.1 | South 24 Pgs, WB |
| RL23 | Gamra/FRV/9-291 | 3.1 ^MR^ | 3.3 ^MR^ | 3.2 ^MR^ | 4.2 ^MR^ | 3.9 ^MR^ | 4.5 ^MR^ | 3.9 ^MR^ | 3.5 ^MR^ | 3.6 ^MR^ | 12.5 | 5.2 | Purulia, WB |
| RL119 | Gangajali/FRV/8-261 | 3.0 ^MR^ | 4.9 ^MR^ | 4.7 ^MR^ | 4.6 ^MR^ | 5.2 ^MS^ | 4.9 ^MR^ | 4.0 ^MR^ | 3.9 ^MR^ | 4.6 ^MR^ | 15.0 | 7.9 | Birbhum, WB |
| RL202 | Garibshal/FRV/5-104 | 4.2 ^MR^ | 5.0 ^MS^ | 3.3 ^MR^ | 3.9 ^MR^ | 5.1 ^MS^ | 4.7 ^MR^ | 4.1 ^MR^ | 3.2 ^MR^ | 3.3 ^MR^ | 17.1 | 7.2 | Purulia, WB |
| RL145 | Gauchi/FRV/9-311 | 8.6 ^S^ | 8.9 ^S^ | 8.2 ^S^ | 9.0 ^HS^ | 8.3 ^S^ | 8.0 ^S^ | 7.9 ^S^ | 8.3 ^S^ | 8.5 ^S^ | 28.9 | 20.3 | Birbhum, WB |
| RL121 | Gheus/FRV/7-201 | 8.8 ^S^ | 7.6 ^S^ | 7.9 ^S^ | 9.0 ^HS^ | 8.1 ^S^ | 8.5 ^S^ | 8.2 ^S^ | 7.7 ^S^ | 8.1 ^S^ | 24.6 | 20.3 | North 24 Pgs, WB |
| RL24 | Gitanjali/FRV/4-014 | 4.2 ^MR^ | 3.8 ^MR^ | 4.5 ^MR^ | 5.3 ^MS^ | 4.9 ^MR^ | 6.2 ^MS^ | 4.2 ^MR^ | 4.0 ^MR^ | 3.7 ^MR^ | 16.8 | 7.8 | South 24 Pgs, WB |
| RL25 | Gobindabhog/FRV/3-010 | 5.2 ^MS^ | 6.4 ^MS^ | 6.0 ^MS^ | 6.3 ^MS^ | 6.8 ^MS^ | 6.5 ^MS^ | 5.8 ^MS^ | 6.0 ^MS^ | 6.5 ^MS^ | 20.2 | 13.6 | Burdwan, WB |
| RL26 | Goradhan/FRV/3-011 | 6.1 ^MS^ | 6.2 ^MS^ | 6.1 ^MS^ | 7.1 ^S^ | 6.5 ^MS^ | 5.8 ^MS^ | 6.2 ^MS^ | 5.8 ^MS^ | 6.6 ^MS^ | 25.6 | 15.5 | East Midnapore, WB |
| RL203 | Gournitai/FRV/6-164 | 5.9 ^MS^ | 6.4 ^MS^ | 6.5 ^MS^ | 5.2 ^MS^ | 7.0 ^S^ | 6.1 ^MS^ | 6.2 ^MS^ | 5.8 ^MS^ | 6.0 ^MS^ | 20.5 | 13.9 | Birbhum, WB |
| RL147 | Gujanonia/FRV/10-347 | 3.9 ^MR^ | 3.6 ^MR^ | 4.8 ^MR^ | 4.9 ^MR^ | 5.1 ^MS^ | 3.8 ^MR^ | 3.9 ^MR^ | 3.6 ^MR^ | 4.3 ^MR^ | 9.8 | 4.9 | South 24 Pgs, WB |
| RL31 | H.M.T./FRV/2-003 | 6.2 ^MS^ | 6.0 ^MS^ | 5.7 ^MS^ | 6.1 ^MS^ | 6.3 ^MS^ | 5.6 ^MS^ | 5.2 ^MS^ | 5.9 ^MS^ | 5.5 ^MS^ | 18.3 | 12.5 | Purulia, WB |
| RL27 | Haldichuri/FRV/4-016 | 2.2 ^R^ | 2.3 ^R^ | 2.0 ^R^ | 1.6 ^R^ | 2.2 ^R^ | 2.5 ^R^ | 1.3 ^R^ | 1.8 ^R^ | 1.5 ^R^ | 7.5 | 1.8 | Birbhum, WB |
| RL28 | Hamai/FRV/5-059 | 8.1 ^S^ | 8.0 ^S^ | 8.6 ^S^ | 9.0 ^HS^ | 9.0 ^HS^ | 9.0 ^HS^ | 8.9 ^S^ | 8.6 ^S^ | 8.2 ^S^ | 31.2 | 21.1 | South 24 Pgs, WB |
| RL204 | Hanumanjata/FRV/6-165 | 3.6 ^MR^ | 4.7 ^MR^ | 4.0 ^MR^ | 4.1 ^MR^ | 4.5 ^MR^ | 5.0 ^MS^ | 3.2 ^MR^ | 3.9 ^MR^ | 3.8 ^MR^ | 10.3 | 4.0 | Birbhum, WB |
| RL29 | Harinkajali/FRV/8-233 | 3.8 ^MR^ | 4.2 ^MR^ | 4.2 ^MR^ | 5.7 ^MS^ | 4.2 ^MR^ | 5.1 ^MS^ | 4.3 ^MR^ | 4.0 ^MR^ | 4.1 ^MR^ | 16.0 | 7.1 | South 24 Pgs, WB |
| RL216 | Harkum/FRV/8-280 | 8.6 ^S^ | 8.0 ^S^ | 8.3 ^S^ | 8.5 ^S^ | 7.6 ^S^ | 9.0 ^HS^ | 8.4 ^S^ | 8.5 ^S^ | 8.3 ^S^ | 27.5 | 20.0 | Birbhum, WB |
| RL30 | Hatidhan/FRV/6-110 | 6.7 ^MS^ | 6.2 ^MS^ | 6.0 ^MS^ | 5.9 ^MS^ | 5.5 ^MS^ | 6.9 ^MS^ | 5.0 ^MS^ | 5.8 ^MS^ | 6.0 ^MS^ | 26.8 | 16.0 | East Midnapore, WB |
| RL205 | Hiramati/FRV/9-326 | 4.5 ^MR^ | 4.1 ^MR^ | 3.9 ^MR^ | 4.6 ^MR^ | 4.9 ^MR^ | 5.1 ^MS^ | 4.1 ^MR^ | 4.0 ^MR^ | 3.6 ^MR^ | 16.4 | 6.8 | South 24 Pgs, WB |
| RL206 | Indrashal/FRV/9-327 | 9.0 ^HS^ | 9.0 ^HS^ | 9.0 ^HS^ | 9.0 ^HS^ | 9.0 ^HS^ | 8.7 ^S^ | 9.0 ^HS^ | 9.0 ^HS^ | 9.0 ^HS^ | 37.2 | 26.2 | Bankura, WB |
| RL32 | Itanagar/FRV/7-177 | 8.2 ^S^ | 7.6 ^S^ | 8.4 ^S^ | 9.0 ^HS^ | 7.2 ^S^ | 9.0 ^HS^ | 8.0 ^S^ | 8.1 ^S^ | 8.4 ^S^ | 28.9 | 19.7 | West Midnapore, WB |
| RL122 | Jaldhepa/FRV/7-205 | 6.2 ^MS^ | 5.7 ^MS^ | 6.0 ^MS^ | 6.5 ^MS^ | 6.4 ^MS^ | 7.2 ^S^ | 6.0 ^MS^ | 6.9 ^MS^ | 5.8 ^MS^ | 14.5 | 11.7 | Coochbehar, WB |
| RL33 | Jalkamini/FRV/6-112 | 4.3 ^MR^ | 5.2 ^MS^ | 4.2 ^MR^ | 5.8 ^MS^ | 3.6 ^MR^ | 4.9 ^MR^ | 4.7 ^MR^ | 4.1 ^MR^ | 4.6 ^MR^ | 14.3 | 8.6 | Birbhum, WB |
| RL34 | Jalthapa/FRV/6-114 | 5.9 ^MS^ | 5.3 ^MS^ | 6.8 ^MS^ | 6.5 ^MS^ | 6.9 ^MS^ | 7.1 ^S^ | 5.8 ^MS^ | 6.2 ^MS^ | 6.9 ^MS^ | 20.3 | 14.1 | South 24 Pgs, WB |
| RL123 | Jamainaru/FRV/9-308 | 3.6 ^MR^ | 3.4 ^MR^ | 4.9 ^MR^ | 4.6 ^MR^ | 5.2 ^MS^ | 5.3 ^MS^ | 4.2 ^MR^ | 3.6 ^MR^ | 4.0 ^MR^ | 14.6 | 6.9 | South 24 Pgs, WB |
| RL35 | Janglijata/FRV/8-235 | 2.3 ^R^ | 2.6 ^R^ | 2.0 ^R^ | 2.9 ^R^ | 1.5 ^R^ | 1.9 ^R^ | 2.2 ^R^ | 1.3 ^R^ | 1.6 ^R^ | 6.8 | 3.0 | East Midnapore, WB |
| RL148 | Jeerashal/FRV/6-145 | 6.2 ^MS^ | 6.0 ^MS^ | 5.1 ^MS^ | 7.2 ^S^ | 5.6 ^MS^ | 6.5 ^MS^ | 5.9 ^MS^ | 5.2 ^MS^ | 6.0 ^MS^ | 26.2 | 14.8 | Purulia, WB |
| RL36 | Jhingeshal/FRV/11-359 | 3.2 ^MR^ | 3.6 ^MR^ | 4.9 ^MR^ | 5.6 ^MS^ | 6.2 ^MS^ | 5.9 ^MS^ | 3.6 ^MR^ | 5.1 ^MS^ | 4.0 ^MR^ | 19.2 | 9.2 | Purulia, WB |
| RL39 | Jhulur/FRV/5-061 | 7.3 ^S^ | 7.8 ^S^ | 7.2 ^S^ | 7.2 ^S^ | 8.6 ^S^ | 8.9 ^S^ | 5.2 ^MS^ | 5.0 ^MS^ | 4.5 ^MR^ | 16.5 | 7.1 | Purulia, WB |
| RL37 | JP-110/FRV/9-293 | 6.2 ^MS^ | 6.1 ^MS^ | 6.4 ^MS^ | 5.9 ^MS^ | 6.2 ^MS^ | 6.6 ^MS^ | 6.0 ^MS^ | 6.1 ^MS^ | 5.3 ^MS^ | 16.2 | 11.3 | East Midnapore, WB |
| RL38 | JP-90/FRV/6-115 | 6.2 ^MS^ | 5.8 ^MS^ | 6.6 ^MS^ | 7.0 ^S^ | 6.8 ^MS^ | 6.5 ^MS^ | 5.8 ^MS^ | 6.2 ^MS^ | 6.0 ^MS^ | 19.9 | 13.7 | Purulia, WB |
| RL40 | Kaike/FRV/5-064 | 5.4 ^MS^ | 6.4 ^MS^ | 6.0 ^MS^ | 6.7 ^MS^ | 5.8 ^MS^ | 7.2 ^S^ | 6.5 ^MS^ | 6.2 ^MS^ | 5.3 ^MS^ | 28.5 | 15.9 | Purulia, WB |
| RL41 | Kakri/FRV/7-180 | 4.2 ^MR^ | 4.8 ^MR^ | 3.9 ^MR^ | 6.8 ^MS^ | 5.9 ^MS^ | 6.5 ^MS^ | 4.2 ^MR^ | 4.8 ^MR^ | 3.9 ^MR^ | 18.9 | 8.8 | Bankura, WB |
| RL42 | Kalabhat/FRV/10-331 | 2.1 ^R^ | 1.6 ^R^ | 1.2 ^R^ | 2.3 ^R^ | 2.6 ^R^ | 2.0 ^R^ | 1.1 ^R^ | 1.4 ^R^ | 1.7 ^R^ | 9.5 | 2.5 | Bankura, WB |
| RL43 | Kalalahi/FRV/11-360 | 6.2 ^MS^ | 6.0 ^MS^ | 6.7 ^MS^ | 6.8 ^MS^ | 6.6 ^MS^ | 5.6 ^MS^ | 5.3 ^MS^ | 5.5 ^MS^ | 5.1 ^MS^ | 22.1 | 13.9 | South 24 Pgs, WB |
| RL124 | Kalamkathi/FRV/11-368 | 4.6 ^MR^ | 4.5 ^MR^ | 4.3 ^MR^ | 5.6 ^MS^ | 5.4 ^MS^ | 6.1 ^MS^ | 4.1 ^MR^ | 3.2 ^MR^ | 3.3 ^MR^ | 20.2 | 10.1 | Bankura, WB |
| RL150 | Kalamogha/FRV/5-090 | 4.1 ^MR^ | 3.9 ^MR^ | 4.6 ^MR^ | 3.9 ^MR^ | 4.5 ^MR^ | 5.0 ^MS^ | 4.4 ^MR^ | 4.1 ^MR^ | 3.6 ^MR^ | 10.0 | 5.6 | South 24 Pgs, WB |
| RL44 | Kalanamak/FRV/9-295 | 3.2 ^MR^ | 3.6 ^MR^ | 3.9 ^MR^ | 4.2 ^MR^ | 4.9 ^MR^ | 4.6 ^MR^ | 3.6 ^MR^ | 4.2 ^MR^ | 4.5 ^MR^ | 12.3 | 6.4 | Purulia, WB |
| RL45 | Kalmugur/FRV/8-237 | 5.9 ^MS^ | 6.8 ^MS^ | 6.6 ^MS^ | 5.2 ^MS^ | 5.8 ^MS^ | 6.1 ^MS^ | 5.2 ^MS^ | 5.5 ^MS^ | 5.9 ^MS^ | 20.6 | 13.5 | Purulia, WB |
| RL46 | Kalochipta/FRV/8-238 | 4.2 ^MR^ | 3.1 ^MR^ | 4.6 ^MR^ | 5.2 ^MS^ | 5.6 ^MS^ | 5.8 ^MS^ | 4.0 ^MR^ | 3.8 ^MR^ | 4.4 ^MR^ | 11.5 | 7.9 | Birbhum, WB |
| RL125 | Kalodhopa/FRV/10-343 | 4.9 ^MR^ | 3.1 ^MR^ | 4.6 ^MR^ | 4.2 ^MR^ | 5.9 ^MS^ | 3.8 ^MR^ | 3.9 ^MR^ | 3.1 ^MR^ | 3.0 ^MR^ | 17.3 | 9.2 | East Midnapore, WB |
| RL162 | Kalojira/FRV/6-150 | 4.2 ^MR^ | 4.8 ^MR^ | 4.7 ^MR^ | 6.1 ^MS^ | 4.2 ^MR^ | 4.5 ^MR^ | 4.6 ^MR^ | 5.0 ^MS^ | 4.7 ^MR^ | 17.0 | 8.7 | Bankura, WB |
| RL126 | Kalokumro/FRV/10-344 | 4.2 ^MR^ | 3.6 ^MR^ | 4.0 ^MR^ | 4.6 ^MR^ | 5.2 ^MS^ | 3.7 ^MR^ | 3.2 ^MR^ | 3.8 ^MR^ | 3.6 ^MR^ | 19.6 | 9.6 | East Midnapore, WB |
| RL47 | Kalomota/FRV/6-117 | 8.0 ^S^ | 7.9 ^S^ | 8.3 ^S^ | 7.5 ^S^ | 8.9 ^S^ | 8.6 ^S^ | 7.6 ^S^ | 7.9 ^S^ | 8.3 ^S^ | 25.3 | 18.2 | South 24 Pgs, WB |
| RL48 | Kalonunia/FRV/5-066 | 5.6 ^MS^ | 5.5 ^MS^ | 4.3 ^MR^ | 6.2 ^MS^ | 4.8 ^MR^ | 4.2 ^MR^ | 4.6 ^MR^ | 4.4 ^MR^ | 4.9 ^MR^ | 11.2 | 8.1 | Jalpaiguri, WB |
| RL49 | Kaltura/FRV/6-119 | 6.2 ^MS^ | 6.8 ^MS^ | 5.8 ^MS^ | 5.9 ^MS^ | 6.6 ^MS^ | 6.9 ^MS^ | 6.2 ^MS^ | 6.0 ^MS^ | 6.1 ^MS^ | 13.2 | 10.6 | West Midnapore, WB |
| RL207 | Kaminibhog/FRV/8-278 | 3.8 ^MR^ | 3.6 ^MR^ | 4.8 ^MR^ | 5.2 ^MS^ | 3.9 ^MR^ | 5.3 ^MS^ | 4.2 ^MR^ | 4.1 ^MR^ | 4.7 ^MR^ | 12.9 | 6.5 | South 24 Pgs, WB |
| RL50 | Kanakchur/FRV/8-240 | 4.0 ^MR^ | 3.0 ^MR^ | 3.5 ^MR^ | 4.8 ^MR^ | 3.9 ^MR^ | 5.0 ^MS^ | 3.6 ^MR^ | 3.8 ^MR^ | 4.0 ^MR^ | 15.8 | 6.8 | South 24 Pgs, WB |
| RL161 | Kankshal/FRV/5-091 | 6.9 ^MS^ | 8.0 ^S^ | 7.7 ^S^ | 8.2 ^S^ | 7.6 ^S^ | 7.8 ^S^ | 7.2 ^S^ | 8.0 ^S^ | 7.7 ^S^ | 25.3 | 17.9 | West Midnapore, WB |
| RL51 | Kantarangi/FRV/6-121 | 5.0 ^MS^ | 4.0 ^MR^ | 3.8 ^MR^ | 4.0 ^MR^ | 5.6 ^MS^ | 6.7 ^MS^ | 4.1 ^MR^ | 4.0 ^MR^ | 4.2 ^MR^ | 14.3 | 7.2 | Purulia, WB |
| RL52 | Kantaribhog/FRV/9-296 | 4.6 ^MR^ | 4.2 ^MR^ | 4.2 ^MR^ | 6.1 ^MS^ | 5.3 ^MS^ | 5.9 ^MS^ | 3.6 ^MR^ | 4.5 ^MR^ | 4.0 ^MR^ | 12.0 | 8.6 | Dinajpur, WB |
| RL54 | Kerala sundari/FRV/10-333 | 3.1 ^MR^ | 3.9 ^MR^ | 3.0 ^MR^ | 4.2 ^MR^ | 3.6 ^MR^ | 5.3 ^MS^ | 4.9 ^MR^ | 4.1 ^MR^ | 4.7 ^MR^ | 9.4 | 4.2 | Purulia, WB |
| RL149 | Kartikshal/FRV/7-212 | 7.9 ^S^ | 7.6 ^S^ | 7.7 ^S^ | 8.9 ^S^ | 9.0 ^HS^ | 8.6 ^S^ | 7.6 ^S^ | 8.0 ^S^ | 7.9 ^S^ | 27.4 | 19.0 | South 24 Pgs, WB |
| RL53 | Kaya/FRV/10-332 | 5.7 ^MS^ | 6.7 ^MS^ | 6.3 ^MS^ | 6.2 ^MS^ | 6.9 ^MS^ | 5.6 ^MS^ | 6.3 ^MS^ | 5.6 ^MS^ | 6.0 ^MS^ | 14.9 | 11.8 | West Midnapore, WB |
| RL55 | Keshabshal/FRV/10-335 | 4.2 ^MR^ | 5.0 ^MS^ | 3.6 ^MR^ | 6.2 ^MS^ | 8.7 ^S^ | 6.0 ^MS^ | 3.7 ^MR^ | 3.6 ^MR^ | 4.0 ^MR^ | 14.2 | 4.1 | Purulia, WB |
| RL56 | Khara/FRV/4-019 | 2.4 ^R^ | 1.7 ^R^ | 2.0 ^R^ | 2.6 ^R^ | 2.9 ^R^ | 1.7 ^R^ | 1.8 ^R^ | 2.3 ^R^ | 1.2 ^R^ | 12.9 | 2.6 | East Midnapore, WB |
| RL57 | Khejurchhari/FRV/5-067 | 4.2 ^MR^ | 3.7 ^MR^ | 4.0 ^MR^ | 4.8 ^MR^ | 5.6 ^MS^ | 5.5 ^MS^ | 3.5 ^MR^ | 3.8 ^MR^ | 3.8 ^MR^ | 20.3 | 9.1 | South 24 Pgs, WB |
| RL127 | Kheuch/FRV/6-141 | 7.6 ^S^ | 8.1 ^S^ | 8.4 ^S^ | 7.7 ^S^ | 8.2 ^S^ | 9.0 ^HS^ | 7.7 ^S^ | 7.9 ^S^ | 8.6 ^S^ | 25.2 | 18.7 | Birbhum, WB |
| RL165 | Khirabichi/FRV/5-093 | 6.7 ^MS^ | 8.6 ^S^ | 8.4 ^S^ | 8.5 ^S^ | 9.0 ^HS^ | 7.9 ^S^ | 8.4 ^S^ | 8.2 ^S^ | 7.9 ^S^ | 31.0 | 20.8 | West Midnapore, WB |
| RL208 | Khudikhasa/FRV/7-217 | 9.0 ^HS^ | 9.0 ^HS^ | 9.0 ^HS^ | 9.0 ^HS^ | 9.0 ^HS^ | 9.0 ^HS^ | 9.0 ^HS^ | 9.0 ^HS^ | 8.9 ^S^ | 34.0 | 25.5 | Birbhum, WB |
| RL58 | Kobirajshal/FRV/4-021 | 4.5 ^MR^ | 4.2 ^MR^ | 4.7 ^MR^ | 5.3 ^MS^ | 4.8 ^MR^ | 6.7 ^MS^ | 7.5 ^S^ | 7.6 ^S^ | 8.2 ^S^ | 13.9 | 20.2 | East Midnapore, WB |
| RL59 | Komal/FRV/6-122 | 6.8 ^MS^ | 6.7 ^MS^ | 6.0 ^MS^ | 7.2 ^S^ | 6.8 ^MS^ | 7.6 ^S^ | 6.7 ^MS^ | 6.9 ^MS^ | 6.5 ^MS^ | 17.3 | 12.1 | East Midnapore, WB |
| RL60 | Koya/FRV/6-126 | 5.8 ^MS^ | 5.9 ^MS^ | 6.3 ^MS^ | 5.9 ^MS^ | 5.7 ^MS^ | 6.9 ^MS^ | 5.7 ^MS^ | 5.5 ^MS^ | 5.0 ^MS^ | 20.2 | 14.3 | Purulia, WB |
| RL61 | Labanshal/FRV/10-336 | 5.6 ^MS^ | 6.8 ^MS^ | 6.1 ^MS^ | 6.1 ^MS^ | 5.9 ^MS^ | 6.7 ^MS^ | 5.8 ^MS^ | 5.2 ^MS^ | 6.0 ^MS^ | 16.9 | 13.4 | East Midnapore, WB |
| RL62 | Lal badshabhog/FRV/10-337 | 4.2 ^MR^ | 4.1 ^MR^ | 3.8 ^MR^ | 5.2 ^MS^ | 5.8 ^MS^ | 4.9 ^MR^ | 3.6 ^MR^ | 4.9 ^MR^ | 4.6 ^MR^ | 12.3 | 6.6 | Purulia, WB |
| RL63 | Lal dudheshwar/FRV/9-298 | 3.0 ^MR^ | 3.3 ^MR^ | 3.6 ^MR^ | 4.6 ^MR^ | 4.5 ^MR^ | 5.0 ^MS^ | 3.2 ^MR^ | 3.6 ^MR^ | 3.5 ^MR^ | 8.7 | 3.9 | South 24 Pgs, WB |
| RL64 | Lalgetu/FRV/7-181 | 5.9 ^MS^ | 6.4 ^MS^ | 6.6 ^MS^ | 6.7 ^MS^ | 7.1 ^S^ | 7.0 ^S^ | 6.2 ^MS^ | 6.6 ^MS^ | 5.9 ^MS^ | 23.5 | 14.0 | Purulia, WB |
| RL163 | Laltipa/FRV/10-351 | 7.8 ^S^ | 8.1 ^S^ | 8.5 ^S^ | 9.0 ^HS^ | 7.2 ^S^ | 7.1 ^S^ | 7.8 ^S^ | 7.6 ^S^ | 8.2 ^S^ | 26.7 | 18.2 | Bankura, WB |
| RL128 | Langalmura/FRV/10-345 | 3.8 ^MR^ | 4.5 ^MR^ | 4.6 ^MR^ | 6.2 ^MS^ | 5.2 ^MS^ | 4.9 ^MR^ | 4.0 ^MR^ | 3.5 ^MR^ | 3.7 ^MR^ | 18.2 | 8.8 | Birbhum, WB |
| RL167 | Latashal/FRV/6-153 | 4.0 ^MR^ | 3.8 ^MR^ | 4.5 ^MR^ | 3.5 ^MR^ | 4.8 ^MR^ | 4.5 ^MR^ | 3.1 ^MR^ | 4.0 ^MR^ | 3.8 ^MR^ | 11.6 | 6.8 | Purulia, WB |
| RL209 | Lilabati/FRV/7-219 | 5.3 ^MS^ | 5.1 ^MS^ | 5.1 ^MS^ | 5.7 ^MS^ | 3.5 ^MR^ | 4.0 ^MR^ | 3.9 ^MR^ | 4.2 ^MR^ | 4.3 ^MR^ | 12.4 | 6.9 | South 24 Pgs, WB |
| RL65 | Loghu/FRV/7-184 | 6.2 ^MS^ | 6.0 ^MS^ | 5.9 ^MS^ | 7.2 ^S^ | 6.3 ^MS^ | 6.7 ^MS^ | 5.8 ^MS^ | 6.6 ^MS^ | 6.2 ^MS^ | 24.4 | 14.9 | East Midnapore, WB |
| RL211 | Mahipal/FRV/6-166 | 6.9 ^MS^ | 5.8 ^MS^ | 6.1 ^MS^ | 5.5 ^MS^ | 7.6 ^S^ | 6.2 ^MS^ | 5.9 ^MS^ | 6.0 ^MS^ | 5.6 ^MS^ | 17.2 | 13.6 | Birbhum, WB |
| RL168 | Mala/FRV/8-270 | 7.0 ^S^ | 7.2 ^S^ | 7.6 ^S^ | 8.2 ^S^ | 8.0 ^S^ | 8.6 ^S^ | 7.7 ^S^ | 7.8 ^S^ | 8.6 ^S^ | 30.6 | 20.1 | West Midnapore, WB |
| RL129 | Malabati/FRV/5-084 | 6.8 ^MS^ | 5.9 ^MS^ | 6.4 ^MS^ | 5.7 ^MS^ | 6.8 ^MS^ | 6.9 ^MS^ | 6.4 ^MS^ | 5.4 ^MS^ | 6.0 ^MS^ | 16.3 | 13.3 | East Midnapore, WB |
| RL66 | Maliaphulo/FRV/11-362 | 7.9 ^S^ | 7.8 ^S^ | 7.1 ^S^ | 6.7 ^MS^ | 7.8 ^S^ | 6.1 ^MS^ | 4.6 ^MR^ | 5.3 ^MS^ | 4.2 ^MR^ | 15.8 | 7.8 | Birbhum, WB |
| RL210 | Mallika/FRV/8-279 | 8.0 ^S^ | 7.2 ^S^ | 7.6 ^S^ | 9.0 ^HS^ | 9.0 ^HS^ | 8.7 ^S^ | 7.8 ^S^ | 8.7 ^S^ | 8.2 ^S^ | 24.6 | 17.9 | South 24 Pgs, WB |
| RL67 | Malshira/FRV/7-185 | 5.7 ^MS^ | 6.8 ^MS^ | 6.4 ^MS^ | 5.8 ^MS^ | 6.8 ^MS^ | 3.2 ^MR^ | 6.0 ^MS^ | 6.1 ^MS^ | 5.7 ^MS^ | 18.3 | 12.3 | Coochbehar, WB |
| RL217 | Maltu/FRV/7-221 | 5.6 ^MS^ | 5.1 ^MS^ | 5.7 ^MS^ | 5.8 ^MS^ | 6.1 ^MS^ | 5.3 ^MS^ | 4.0 ^MR^ | 4.9 ^MR^ | 4.6 ^MR^ | 21.8 | 10.1 | Birbhum, WB |
| RL169 | Manik kalam/FRV/9-315 | 7.4 ^S^ | 8.2 ^S^ | 8.8 ^S^ | 7.3 ^S^ | 7.6 ^S^ | 8.7 ^S^ | 8.2 ^S^ | 8.1 ^S^ | 8.4 ^S^ | 26.2 | 19.5 | Bankura, WB |
| RL68 | Marichshal/FRV/8-241 | 3.7 ^MR^ | 4.6 ^MR^ | 4.7 ^MR^ | 4.8 ^MR^ | 6.5 ^MS^ | 5.2 ^MS^ | 4.2 ^MR^ | 3.3 ^MR^ | 3.9 ^MR^ | 10.8 | 4.7 | South 24 Pgs, WB |
| RL69 | Mashlot/FRV/8-243 | 6.2 ^MS^ | 6.8 ^MS^ | 6.9 ^MS^ | 6.1 ^MS^ | 5.5 ^MS^ | 6.0 ^MS^ | 5.6 ^MS^ | 5.3 ^MS^ | 5.8 ^MS^ | 15.9 | 10.8 | East Midnapore, WB |
| RL70 | Medi/FRV/4-026 | 4.0 ^MR^ | 4.6 ^MR^ | 3.8 ^MR^ | 4.2 ^MR^ | 6.1 ^MS^ | 4.8 ^MR^ | 3.6 ^MR^ | 3.0 ^MR^ | 4.0 ^MR^ | 18.7 | 5.9 | South 24 Pgs, WB |
| RL71 | Meghi/FRV/3-008 | 5.7 ^MS^ | 6.2 ^MS^ | 6.4 ^MS^ | 6.9 ^MS^ | 7.3 ^S^ | 5.9 ^MS^ | 6.1 ^MS^ | 6.2 ^MS^ | 5.8 ^MS^ | 16.1 | 11.6 | Bankura, WB |
| RL72 | Meghnadomru/FRV/4-027 | 4.8 ^MR^ | 5.5 ^MS^ | 5.8 ^MS^ | 4.5 ^MR^ | 5.6 ^MS^ | 5.1 ^MS^ | 5.2 ^MS^ | 4.7 ^MR^ | 4.5 ^MR^ | 12.7 | 9.3 | Bankura, WB |
| RL73 | Megi/FRV/8-250 | 6.2 ^MS^ | 6.1 ^MS^ | 6.6 ^MS^ | 6.1 ^MS^ | 5.7 ^MS^ | 6.3 ^MS^ | 5.9 ^MS^ | 5.1 ^MS^ | 5.3 ^MS^ | 20.7 | 14.4 | Birbhum, WB |
| RL130 | Mihidana/FRV/7-206 | 3.8 ^MR^ | 3.2 ^MR^ | 4.2 ^MR^ | 6.3 ^MS^ | 6.0 ^MS^ | 5.5 ^MS^ | 8.0 ^S^ | 7.9 ^S^ | 8.6 ^S^ | 22.6 | 20.5 | Purulia, WB |
| RL74 | Mohanbhog/FRV/11-363 | 5.3 ^MS^ | 5.8 ^MS^ | 5.7 ^MS^ | 6.4 ^MS^ | 7.5 ^S^ | 6.7 ^MS^ | 6.1 ^MS^ | 6.8 ^MS^ | 6.6 ^MS^ | 24.1 | 15.1 | Purulia, WB |
| RL75 | Moulo/FRV/6-128 | 5.6 ^MS^ | 6.2 ^MS^ | 6.0 ^MS^ | 6.5 ^MS^ | 7.0 ^S^ | 6.2 ^MS^ | 6.5 ^MS^ | 5.2 ^MS^ | 5.4 ^MS^ | 14.6 | 13.9 | South 24 Pgs, WB |
| RL76 | Mugai/FRV/6-129 | 5.2 ^MS^ | 3.9 ^MR^ | 3.8 ^MR^ | 6.7 ^MS^ | 5.0 ^MS^ | 6.4 ^MS^ | 4.6 ^MR^ | 4.5 ^MR^ | 3.9 ^MR^ | 16.3 | 9.1 | West Midnapore, WB |
| RL77 | Mugi/FRV/7-186 | 4.5 ^MR^ | 4.9 ^MR^ | 3.1 ^MR^ | 4.2 ^MR^ | 5.7 ^MS^ | 5.0 ^MS^ | 3.6 ^MR^ | 4.0 ^MR^ | 3.7 ^MR^ | 11.9 | 8.3 | Purulia, WB |
| RL151 | Mugurshal/FRV/9-312 | 8.0 ^S^ | 7.6 ^S^ | 8.3 ^S^ | 8.2 ^S^ | 8.1 ^S^ | 9.0 ^HS^ | 7.6 ^S^ | 7.7 ^S^ | 7.5 ^S^ | 26.1 | 18.8 | East Midnapore, WB |
| RL131 | Mukta/FRV/10-346 | 4.2 ^MR^ | 3.6 ^MR^ | 3.9 ^MR^ | 4.6 ^MR^ | 5.0 ^MS^ | 4.9 ^MR^ | 3.6 ^MR^ | 3.0 ^MR^ | 3.9 ^MR^ | 11.3 | 6.1 | Purulia, WB |
| RL170 | Murgibalam/FRV/9-316 | 8.2 ^S^ | 7.2 ^S^ | 7.4 ^S^ | 5.5 ^MS^ | 7.7 ^S^ | 7.0 ^S^ | 4.7 ^MR^ | 4.7 ^MR^ | 4.9 ^MR^ | 11.5 | 6.5 | Bankura, WB |
| RL212 | Nabannashal/FRV/6-167 | 6.2 ^MS^ | 6.6 ^MS^ | 6.5 ^MS^ | 6.4 ^MS^ | 7.1 ^S^ | 7.0 ^S^ | 6.2 ^MS^ | 6.7 ^MS^ | 6.0 ^MS^ | 21.5 | 14.5 | Bankura, WB |
| RL78 | Nagaland/FRV/7-189 | 6.1 ^MS^ | 5.9 ^MS^ | 6.6 ^MS^ | 7.0 ^S^ | 6.2 ^MS^ | 5.6 ^MS^ | 5.9 ^MS^ | 6.3 ^MS^ | 6.7 ^MS^ | 17.2 | 11.2 | West Midnapore, WB |
| RL79 | Nageshwari/FRV/9-300 | 6.5 ^MS^ | 6.7 ^MS^ | 5.5 ^MS^ | 5.2 ^MS^ | 6.3 ^MS^ | 6.4 ^MS^ | 6.6 ^MS^ | 5.5 ^MS^ | 6.2 ^MS^ | 21.5 | 13.1 | Birbhum, WB |
| RL152 | Nagradhan/FRV/10-348 | 7.6 ^S^ | 7.1 ^S^ | 7.9 ^S^ | 6.1 ^MS^ | 7.5 ^S^ | 5.6 ^MS^ | 5.1 ^MS^ | 4.6 ^MR^ | 4.8 ^MR^ | 18.1 | 7.6 | West Midnapore, WB |
| RL80 | Nagrapatnai/FRV/11-364 | 4.1 ^MR^ | 4.2 ^MR^ | 4.6 ^MR^ | 5.9 ^MS^ | 5.6 ^MS^ | 6.2 ^MS^ | 4.8 ^MR^ | 4.2 ^MR^ | 4.5 ^MR^ | 14.0 | 7.7 | Hooghly, WB |
| RL132 | Nagrashal/FRV/4-039 | 4.8 ^MR^ | 3.2 ^MR^ | 3.8 ^MR^ | 5.2 ^MS^ | 6.1 ^MS^ | 5.7 ^MS^ | 3.2 ^MR^ | 4.0 ^MR^ | 3.6 ^MR^ | 13.9 | 6.8 | Purulia, WB |
| RL81 | Narayankamini/FRV/10-338 | 4.2 ^MR^ | 4.3 ^MR^ | 4.8 ^MR^ | 6.1 ^MS^ | 4.9 ^MR^ | 5.2 ^MS^ | 6.9 ^MS^ | 8.1 ^S^ | 7.5 ^S^ | 11.5 | 19.6 | Birbhum, WB |
| RL215 | Nayanmani/FRV/2-004 | 5.8 ^MS^ | 6.2 ^MS^ | 6.4 ^MS^ | 7.2 ^S^ | 5.0 ^MS^ | 6.3 ^MS^ | 5.8 ^MS^ | 5.6 ^MS^ | 6.1 ^MS^ | 14.6 | 12.9 | Nadia, WB |
| RL133 | Neta/FRV/5-085 | 8.1 ^S^ | 8.6 ^S^ | 8.5 ^S^ | 7.1 ^S^ | 9.0 ^HS^ | 8.6 ^S^ | 7.2 ^S^ | 7.6 ^S^ | 7.5 ^S^ | 25.0 | 18.3 | East Midnapore, WB |
| RL134 | Nikunja/FRV/4-042 | 3.7 ^MR^ | 4.0 ^MR^ | 4.4 ^MR^ | 4.6 ^MR^ | 5.7 ^MS^ | 5.5 ^MS^ | 4.0 ^MR^ | 4.1 ^MR^ | 3.9 ^MR^ | 16.5 | 8.3 | South 24 Pgs, WB |
| RL82 | Noichi/FRV/9-301 | 3.3 ^MR^ | 3.9 ^MR^ | 4.8 ^MR^ | 4.6 ^MR^ | 3.9 ^MR^ | 4.1 ^MR^ | 3.9 ^MR^ | 4.0 ^MR^ | 3.8 ^MR^ | 16.3 | 6.6 | West Midnapore, WB |
| RL135 | Nonabagra/FRV/3-007 | 8.6 ^S^ | 9.0 ^HS^ | 9.0 ^HS^ | 9.0 ^HS^ | 8.8 ^S^ | 9.0 ^HS^ | 8.6 ^S^ | 9.0 ^HS^ | 9.0 ^HS^ | 35.7 | 23.8 | South 24 Pgs, WB |
| RL173 | Paan/FRV/8-271 | 9.0 ^HS^ | 9.0 ^HS^ | 9.0 ^HS^ | 8.1 ^S^ | 9.0 ^HS^ | 8.6 ^S^ | 9.0 ^HS^ | 9.0 ^HS^ | 9.0 ^HS^ | 34.6 | 23.8 | Birbhum, WB |
| RL171 | Padmashal/FRV/9-317 | 4.9 ^MR^ | 3.9 ^MR^ | 4.0 ^MR^ | 8.2 ^S^ | 5.2 ^MS^ | 5.7 ^MS^ | 3.6 ^MR^ | 3.9 ^MR^ | 4.2 ^MR^ | 18.2 | 5.4 | Birbhum, WB |
| RL172 | Panchali/FRV/10-352 | 6.2 ^MS^ | 5.6 ^MS^ | 5.9 ^MS^ | 6.7 ^MS^ | 7.2 ^S^ | 5.6 ^MS^ | 5.8 ^MS^ | 6.2 ^MS^ | 6.4 ^MS^ | 24.6 | 14.6 | Bankura, WB |
| RL83 | Pari/FRV/9-302 | 5.8 ^MS^ | 5.9 ^MS^ | 6.7 ^MS^ | 7.1 ^S^ | 6.4 ^MS^ | 6.8 ^MS^ | 6.3 ^MS^ | 5.8 ^MS^ | 6.0 ^MS^ | 20.2 | 13.1 | West Midnapore, WB |
| RL84 | Pateni/FRV/7-195 | 6.8 ^MS^ | 6.2 ^MS^ | 6.3 ^MS^ | 5.7 ^MS^ | 5.2 ^MS^ | 7.4 ^S^ | 6.2 ^MS^ | 5.9 ^MS^ | 5.5 ^MS^ | 13.9 | 10.2 | North 24 Pgs, WB |
| RL85 | Patikalam/FRV/7-196 | 5.2 ^MS^ | 3.1 ^MR^ | 4.5 ^MR^ | 6.6 ^MS^ | 6.9 ^MS^ | 5.9 ^MS^ | 3.5 ^MR^ | 3.8 ^MR^ | 4.0 ^MR^ | 15.2 | 6.2 | East Midnapore, WB |
| RL86 | Patnai-23/FRV/7-198 | 6.8 ^MS^ | 6.2 ^MS^ | 6.3 ^MS^ | 5.7 ^MS^ | 6.2 ^MS^ | 6.5 ^MS^ | 5.6 ^MS^ | 6.2 ^MS^ | 6.0 ^MS^ | 14.6 | 12.2 | South 24 Pgs, WB |
| RL160 | Phulkhar/FRV/4-044 | 5.8 ^MS^ | 6.8 ^MS^ | 5.6 ^MS^ | 6.9 ^MS^ | 7.2 ^S^ | 7.7 ^S^ | 5.2 ^MS^ | 6.7 ^MS^ | 6.5 ^MS^ | 21.7 | 15.0 | Birbhum, WB |
| RL120 | Phulpagri/FRV/8-262 | 8.9 ^S^ | 7.8 ^S^ | 8.0 ^S^ | 8.0 ^S^ | 7.9 ^S^ | 9.0 ^HS^ | 7.6 ^S^ | 5.7 ^MS^ | 8.0 ^S^ | 28.1 | 19.5 | Coochbehar, WB |
| RL153 | Purnima/FRV/10-350 | 8.1 ^S^ | 7.9 ^S^ | 7.5 ^S^ | 7.2 ^S^ | 7.9 ^S^ | 8.5 ^S^ | 8.2 ^S^ | 8.8 ^S^ | 8.6 ^S^ | 44.5 | 17.1 | Birbhum, WB |
| RL213 | Radhashree/FRV/4-055 | 7.8 ^S^ | 8.5 ^S^ | 8.5 ^S^ | 7.6 ^S^ | 8.9 ^S^ | 8.9 ^S^ | 8.1 ^S^ | 7.9 ^S^ | 7.9 ^S^ | 27.3 | 19.6 | Purulia, WB |
| RL87 | Radhatilak/FRV/8-251 | 5.8 ^MS^ | 5.4 ^MS^ | 6.0 ^MS^ | 8.0 ^S^ | 5.9 ^MS^ | 6.8 ^MS^ | 6.1 ^MS^ | 6.9 ^MS^ | 6.6 ^MS^ | 18.1 | 12.7 | East Midnapore, WB |
| RL88 | Radhunipagal/FRV/6-132 | 3.2 ^MR^ | 5.0 ^MS^ | 4.0 ^MR^ | 4.6 ^MR^ | 4.9 ^MR^ | 3.8 ^MR^ | 4.2 ^MR^ | 3.6 ^MR^ | 4.0 ^MR^ | 7.8 | 5.0 | Birbhum, WB |
| RL136 | Raghushal/FRV/3-005 | 4.1 ^MR^ | 3.9 ^MR^ | 3.7 ^MR^ | 4.2 ^MR^ | 6.5 ^MS^ | 6.0 ^MS^ | 5.1 ^MS^ | 3.9 ^MR^ | 4.1 ^MR^ | 17.5 | 8.8 | Birbhum, WB |
| RL214 | Rajlaxmi/FRV/5-106 | 7.0 ^S^ | 7.9 ^S^ | 7.5 ^S^ | 6.8 ^MS^ | 6.6 ^MS^ | 7.0 ^S^ | 4.6 ^MR^ | 4.7 ^MR^ | 4.5 ^MR^ | 17.5 | 7.0 | Purulia, WB |
| RL89 | Ramchandrabhog/FRV/5-069 | 4.8 ^MR^ | 5.9 ^MS^ | 6.7 ^MS^ | 7.3 ^S^ | 6.1 ^MS^ | 7.1 ^S^ | 6.2 ^MS^ | 5.8 ^MS^ | 6.0 ^MS^ | 19.9 | 13.1 | Birbhum, WB |
| RL90 | Ramlichonch/FRV/5-070 | 4.1 ^MR^ | 4.6 ^MR^ | 3.8 ^MR^ | 3.2 ^MR^ | 4.1 ^MR^ | 4.0 ^MR^ | 4.6 ^MR^ | 3.9 ^MR^ | 4.5 ^MR^ | 12.6 | 7.3 | Bankura, WB |
| RL91 | Ranibeli/FRV/6-133 | 5.9 ^MS^ | 6.3 ^MS^ | 6.8 ^MS^ | 5.9 ^MS^ | 6.2 ^MS^ | 6.8 ^MS^ | 6.3 ^MS^ | 5.8 ^MS^ | 5.6 ^MS^ | 20.2 | 14.6 | Bankura, WB |
| RL92 | Ranikajal/FRV/6-135 | 6.1 ^MS^ | 6.0 ^MS^ | 6.5 ^MS^ | 7.1 ^S^ | 6.8 ^MS^ | 7.0 ^S^ | 5.2 ^MS^ | 6.7 ^MS^ | 6.5 ^MS^ | 12.6 | 10.3 | East Midnapore, WB |
| RL93 | Rupshal/FRV/8-252 | 5.1 ^MS^ | 5.6 ^MS^ | 6.9 ^MS^ | 5.7 ^MS^ | 7.1 ^S^ | 6.2 ^MS^ | 5.3 ^MS^ | 5.4 ^MS^ | 5.2 ^MS^ | 12.9 | 11.0 | South 24 Pgs, WB |
| RL94 | Sabita/FRV/10-339 | 5.8 ^MS^ | 6.2 ^MS^ | 6.3 ^MS^ | 6.7 ^MS^ | 6.8 ^MS^ | 7.4 ^S^ | 6.0 ^MS^ | 6.5 ^MS^ | 6.1 ^MS^ | 18.3 | 13.1 | South 24 Pgs, WB |
| RL95 | Sadajetu/FRV/5-073 | 6.1 ^MS^ | 5.6 ^MS^ | 5.9 ^MS^ | 5.5 ^MS^ | 7.2 ^S^ | 6.5 ^MS^ | 5.9 ^MS^ | 5.4 ^MS^ | 5.7 ^MS^ | 24.5 | 15.2 | Birbhum, WB |
| RL96 | Sadamota/FRV/5-075 | 5.8 ^MS^ | 6.4 ^MS^ | 6.5 ^MS^ | 7.8 ^S^ | 7.1 ^S^ | 5.3 ^MS^ | 6.1 ^MS^ | 6.8 ^MS^ | 5.9 ^MS^ | 19.1 | 14.3 | South 24 Pgs, WB |
| RL97 | Safari/FRV/4-029 | 6.2 ^MS^ | 6.1 ^MS^ | 6.0 ^MS^ | 6.3 ^MS^ | 7.1 ^S^ | 6.6 ^MS^ | 5.2 ^MS^ | 5.9 ^MS^ | 6.3 ^MS^ | 18.8 | 13.5 | East Midnapore, WB |
| RL99 | Satia/FRV/4-032 | 8.2 ^S^ | 7.1 ^S^ | 7.3 ^S^ | 5.2 ^MS^ | 7.2 ^S^ | 7.9 ^S^ | 4.8 ^MR^ | 4.9 ^MR^ | 4.8 ^MR^ | 25.1 | 8.8 | West Midnapore, WB |
| RL218 | Satin/FRV/6-170 | 8.9 ^S^ | 9.0 ^HS^ | 9.0 ^HS^ | 8.3 ^S^ | 9.0 ^HS^ | 9.0 ^HS^ | 9.0 ^HS^ | 9.0 ^HS^ | 9.0 ^HS^ | 33.1 | 24.2 | South 24 Pgs, WB |
| RL98 | Shalkele/FRV/4-031 | 4.2 ^MR^ | 3.3 ^MR^ | 3.7 ^MR^ | 5.6 ^MS^ | 4.8 ^MR^ | 5.2 ^MS^ | 3.2 ^MR^ | 4.0 ^MR^ | 3.5 ^MR^ | 16.2 | 5.8 | Birbhum, WB |
| RL100 | Shiuli/FRV/4-037 | 3.9 ^MR^ | 5.2 ^MS^ | 4.6 ^MR^ | 6.9 ^MS^ | 6.2 ^MS^ | 5.7 ^MS^ | 4.6 ^MR^ | 4.0 ^MR^ | 4.2 ^MR^ | 12.7 | 6.4 | West Midnapore, WB |
| RL178 | Shotput/FRV/10-353 | 3.6 ^MR^ | 4.7 ^MR^ | 4.0 ^MR^ | 6.2 ^MS^ | 5.7 ^MS^ | 5.8 ^MS^ | 4.2 ^MR^ | 4.6 ^MR^ | 4.9 ^MR^ | 13.8 | 6.0 | Bankura, WB |
| RL137 | Sindurmukhi/FRV/8-263 | 4.6 ^MR^ | 4.7 ^MR^ | 4.7 ^MR^ | 6.1 ^MS^ | 5.2 ^MS^ | 5.9 ^MS^ | 3.6 ^MR^ | 3.5 ^MR^ | 4.0 ^MR^ | 15.1 | 7.5 | Bankura, WB |
| RL138 | Sita/FRV/5-088 | 9.0 ^HS^ | 8.6 ^S^ | 7.2 ^S^ | 8.2 ^S^ | 9.0 ^HS^ | 9.0 ^HS^ | 7.9 ^S^ | 8.0 ^S^ | 8.3 ^S^ | 25.5 | 19.1 | West Midnapore, WB |
| RL101 | Sitabhog/FRV/8-254 | 5.9 ^MS^ | 6.4 ^MS^ | 6.7 ^MS^ | 6.4 ^MS^ | 7.2 ^S^ | 6.8 ^MS^ | 5.4 ^MS^ | 6.2 ^MS^ | 6.0 ^MS^ | 20.7 | 14.1 | Bankura, WB |
| RL139 | Sitashal/FRV/6-142 | 7.5 ^S^ | 8.2 ^S^ | 8.1 ^S^ | 9.0 ^HS^ | 8.3 ^S^ | 9.0 ^HS^ | 8.2 ^S^ | 8.1 ^S^ | 7.6 ^S^ | 29.9 | 19.9 | West Midnapore, WB |
| RL179 | Sonajhuli/FRV/9-319 | 8.1 ^S^ | 8.9 ^S^ | 8.2 ^S^ | 7.5 ^S^ | 8.2 ^S^ | 7.3 ^S^ | 7.3 ^S^ | 7.5 ^S^ | 7.5 ^S^ | 27.1 | 18.0 | Birbhum, WB |
| RL102 | Sonali/FRV/8-255 | 5.2 ^MS^ | 6.2 ^MS^ | 6.0 ^MS^ | 5.6 ^MS^ | 6.8 ^MS^ | 6.2 ^MS^ | 5.1 ^MS^ | 5.2 ^MS^ | 5.0 ^MS^ | 22.3 | 14.5 | Purulia, WB |
| RL103 | Sonasari/FRV/9-304 | 3.0 ^MR^ | 3.9 ^MR^ | 4.9 ^MR^ | 4.2 ^MR^ | 5.8 ^MS^ | 4.6 ^MR^ | 4.2 ^MR^ | 4.0 ^MR^ | 4.1 ^MR^ | 14.8 | 7.1 | Birbhum, WB |
| RL140 | Suakalma/FRV/7-209 | 3.6 ^MR^ | 4.5 ^MR^ | 4.7 ^MR^ | 5.6 ^MS^ | 5.8 ^MS^ | 6.3 ^MS^ | 4.1 ^MR^ | 3.2 ^MR^ | 3.9 ^MR^ | 14.8 | 6.2 | Bankura, WB |
| RL104 | Sundari/FRV/6-137 | 6.7 ^MS^ | 6.6 ^MS^ | 6.8 ^MS^ | 6.8 ^MS^ | 7.2 ^S^ | 5.9 ^MS^ | 6.1 ^MS^ | 6.3 ^MS^ | 6.6 ^MS^ | 16.9 | 14.0 | East Midnapore, WB |
| RL175 | Swapandali/FRV/6-159 | 4.6 ^MR^ | 3.2 ^MR^ | 4.8 ^MR^ | 4.9 ^MR^ | 6.8 ^MS^ | 6.5 ^MS^ | 5.2 ^MS^ | 3.9 ^MR^ | 3.6 ^MR^ | 20.1 | 9.3 | Purulia, WB |
| RL141 | Talmugur/FRV/9-310 | 4.2 ^MR^ | 4.0 ^MR^ | 4.3 ^MR^ | 4.9 ^MR^ | 6.1 ^MS^ | 5.5 ^MS^ | 3.7 ^MR^ | 4.7 ^MR^ | 4.0 ^MR^ | 9.3 | 5.6 | South 24 Pgs, WB |
| RL105 | Talmuli/FRV/5-079 | 4.2 ^MR^ | 4.8 ^MR^ | 5.0 ^MS^ | 5.2 ^MS^ | 6.1 ^MS^ | 5.4 ^MS^ | 3.8 ^MR^ | 3.2 ^MR^ | 3.5 ^MR^ | 19.2 | 8.2 | Birbhum, WB |
| RL106 | Tangrashal/FRV/5-082 | 3.8 ^MR^ | 4.0 ^MR^ | 4.4 ^MR^ | 4.3 ^MR^ | 4.8 ^MR^ | 5.5 ^MS^ | 4.8 ^MR^ | 4.9 ^MR^ | 4.5 ^MR^ | 18.9 | 9.0 | Purulia, WB |
| RL154 | Tejasili/FRV/8-267 | 7.3 ^S^ | 8.6 ^S^ | 8.0 ^S^ | 8.6 ^S^ | 8.2 ^S^ | 7.7 ^S^ | 7.2 ^S^ | 7.9 ^S^ | 8.1 ^S^ | 25.8 | 17.6 | Purulia, WB |
| RL107 | Thubi/FRV/10-341 | 5.9 ^MS^ | 5.8 ^MS^ | 6.7 ^MS^ | 6.5 ^MS^ | 7.8 ^S^ | 7.1 ^S^ | 6.2 ^MS^ | 6.8 ^MS^ | 6.0 ^MS^ | 25.5 | 15.1 | Birbhum, WB |
| RL174 | Tikarnadi/FRV/6-157 | 8.4 ^S^ | 8.0 ^S^ | 8.7 ^S^ | 8.3 ^S^ | 7.6 ^S^ | 8.9 ^S^ | 7.4 ^S^ | 7.6 ^S^ | 7.2 ^S^ | 25.3 | 16.9 | Purulia, WB |
| RL108 | Tilak kachhari/FRV/9-305 | 7.2 ^S^ | 7.9 ^S^ | 8.0 ^S^ | 8.2 ^S^ | 8.9 ^S^ | 9.0 ^HS^ | 7.4 ^S^ | 7.6 ^S^ | 8.0 ^S^ | 30.0 | 20.2 | Murshidabad, WB |
| RL109 | Tulaipanji/FRV/6-138 | 5.2 ^MS^ | 5.1 ^MS^ | 6.1 ^MS^ | 6.1 ^MS^ | 5.3 ^MS^ | 4.7 ^MR^ | 5.0 ^MS^ | 4.1 ^MR^ | 4.6 ^MR^ | 18.3 | 10.3 | Dinajpur, WB |
| RL142 | Tulsibhog/FRV/6-144 | 5.2 ^MS^ | 5.5 ^MS^ | 5.9 ^MS^ | 5.2 ^MS^ | 6.1 ^MS^ | 5.8 ^MS^ | 4.4 ^MR^ | 4.2 ^MR^ | 4.8 ^MR^ | 20.9 | 10.5 | Purulia, WB |
| RL110 | Tulsimukul/FRV/7-200 | 3.9 ^MR^ | 4.6 ^MR^ | 4.5 ^MR^ | 4.6 ^MR^ | 5.8 ^MS^ | 4.9 ^MR^ | 4.0 ^MR^ | 4.9 ^MR^ | 4.5 ^MR^ | 11.6 | 6.1 | Purulia, WB |
| RL111 | Yugal/FRV/8-258 | 3.6 ^MR^ | 3.9 ^MR^ | 4.0 ^MR^ | 5.2 ^MS^ | 4.6 ^MR^ | 5.0 ^MS^ | 3.6 ^MR^ | 3.6 ^MR^ | 3.9 ^MR^ | 17.8 | 8.0 | Birbhum, WB |
| RL112 | Zinni/FRV/6-140 | 4.6 ^MR^ | 4.3 ^MR^ | 3.9 ^MR^ | 5.3^MS^ | 6.1 ^MS^ | 3.8 ^MR^ | 4.2 ^MR^ | 3.8 ^MR^ | 4.3 ^MR^ | 20.0 | 9.3 | West Midnapore, WB |
| SC(S) | Swarna (MTU 7029) | 9.0 ^HS^ | 9.0 ^HS^ | 9.0 ^HS^ | 9.0 ^HS^ | 9.0 ^HS^ | 9.0 ^HS^ | 9.0 ^HS^ | 9.0 ^HS^ | 9.0 ^HS^ | 36.5 | 34.8 | NRRI, Cuttak, Odisha |
| SC(R) | Ptb 33 | 1.2 ^R^ | 2.4 ^R^ | 2.4 ^R^ | 2.3 ^R^ | 1.6 ^R^ | 2.8 ^R^ | 2.1 ^R^ | 1.4 ^R^ | 1.9 ^R^ | 9.7 | 2.6 | NRRI, Cuttak, Odisha |

*Designations of the rice landraces provided by Directorate of Agriculture, Govt. of West Bengal, 63 N.S. Road, Kolkata, India

RL: Rice landrace; SC(S): Standard check (Susceptible); SC(R): Standard check (Resistant)

R: Resistant; MR: Moderately Resistant; MS: Moderately Susceptible; S: Susceptible; HS: Highly Susceptible; WB: West Bengal; NRRI: National Rice Research Institute

**Table S2:** Total variance explained for each component based on different feeding attributing factors of BPH on selected rice landraces

| **Healthy rice landraces** | | | | | | | | | |
| --- | --- | --- | --- | --- | --- | --- | --- | --- | --- |
|  | **Factors** | | | | | | | | |
|  | **F1** | **F2** | **F3** | **F4** | **F5** | **F6** | **F7** | **F8** | **F9** |
| Eigenvalue | 4.352 | 1.268 | 1.029 | 0.823 | 0.460 | 0.426 | 0.400 | 0.138 | 0.105 |
| Variability (%) | 48.351 | 14.084 | 11.437 | 9.147 | 5.114 | 4.728 | 4.442 | 1.535 | 1.162 |
| Cumulative (%) | 48.351 | 62.435 | 73.872 | 83.019 | 88.133 | 92.861 | 97.303 | 98.838 | 100.00 |
| **BPH infested rice landraces** | | | | | | | | | |
|  | **Factors** | | | | | | | | |
|  | **F1** | **F2** | **F3** | **F4** | **F5** | **F6** | **F7** | **F8** | **F9** |
| Eigenvalue | 4.400 | 1.213 | 1.043 | 0.852 | 0.585 | 0.350 | 0.272 | 0.169 | 0.115 |
| Variability (%) | 48.891 | 13.473 | 11.593 | 9.467 | 6.502 | 3.893 | 3.022 | 1.879 | 1.280 |
| Cumulative (%) | 48.891 | 62.364 | 73.957 | 83.424 | 89.926 | 93.819 | 96.840 | 98.720 | 100.000 |

F1: Nitrogen; F2: Total Phenol; F3: Phosphorus, F4: Potassium; F5: Reducing sugar, F6: Ascorbic acid; F7: Oxalic acid; F8: Crude silica; F9: Total free amino acid

**Table S3:** Principal factor matrix after varimax rotation (Kaiser Normalization) for different feeding attributing factors of BPH on selected rice landraces

| **Healthy rice landraces** | | | | | | | | | | |
| --- | --- | --- | --- | --- | --- | --- | --- | --- | --- | --- |
| **Parameters** | **Unit** | **Factors** | | | | | | | | |
|  |  | **F1** | **F2** | **F3** | **F4** | **F5** | **F6** | **F7** | **F8** | **F9** |
| Nitrogen (N) | % | **0.702** | 0.025 | 0.000 | 0.011 | 0.017 | 0.021 | 0.191 | 0.033 | 0.000 |
| Total phenol (TP) | mg g^-1^ tissue | **0.521** | 0.063 | 0.003 | 0.250 | 0.008 | 0.123 | 0.011 | 0.010 | 0.013 |
| Phosphorus (P) | % | 0.175 | 0.131 | 0.274 | **0.367** | 0.000 | 0.013 | 0.038 | 0.002 | 0.000 |
| Potassium (K) | % | 0.045 | **0.583** | 0.259 | 0.004 | 0.082 | 0.017 | 0.006 | 0.002 | 0.003 |
| Reducing sugar (RS) | mg g^-1^ of glucose equivalent | **0.817** | 0.004 | 0.005 | 0.009 | 0.056 | 0.036 | 0.002 | 0.039 | 0.031 |
| Ascorbic acid (AS) | mg g^-1^ tissue | 0.180 | 0.105 | **0.484** | 0.135 | 0.029 | 0.050 | 0.014 | 0.002 | 0.002 |
| Oxalic acid (OA) | mg g^-1^ tissue | **0.493** | 0.300 | 0.004 | 0.000 | 0.152 | 0.007 | 0.013 | 0.015 | 0.016 |
| Crude silica (CS) | % | **0.604** | 0.012 | 0.000 | 0.047 | 0.113 | 0.114 | 0.108 | 0.000 | 0.001 |
| Total free amino acid (TFA) | µg g^-1^ glutamic acid equivalent | **0.815** | 0.045 | 0.001 | 0.002 | 0.003 | 0.045 | 0.017 | 0.035 | 0.039 |
| **BPH infested rice landraces** | | | | | | | | | | |
| **Parameters** | **Unit** | **Factors** | | | | | | | | |
|  |  | **F1** | **F2** | **F3** | **F4** | **F5** | **F6** | **F7** | **F8** | **F9** |
| Nitrogen (N) | % | **0.695** | 0.019 | 0.001 | 0.003 | 0.073 | 0.169 | 0.014 | 0.023 | 0.003 |
| Total phenol (TP) | mg g^-1^ tissue | 0.065 | **0.698** | 0.112 | 0.021 | 0.083 | 0.004 | 0.003 | 0.004 | 0.010 |
| Phosphorus (P) | % | 0.173 | 0.252 | 0.000 | **0.443** | 0.129 | 0.001 | 0.000 | 0.001 | 0.001 |
| Potassium (K) | % | 0.026 | 0.094 | **0.832** | 0.005 | 0.022 | 0.010 | 0.006 | 0.004 | 0.000 |
| Reducing sugar (RS) | mg g^-1^ of glucose equivalent | **0.828** | 0.028 | 0.036 | 0.002 | 0.002 | 0.013 | 0.000 | 0.027 | 0.063 |
| Ascorbic acid (AS) | mg g^-1^ tissue | **0.828** | 0.005 | 0.000 | 0.001 | 0.036 | 0.002 | 0.045 | 0.054 | 0.030 |
| Oxalic acid (OA) | mg g^-1^ tissue | 0.304 | 0.097 | 0.003 | **0.366** | 0.223 | 0.003 | 0.004 | 0.000 | 0.002 |
| Crude silica (CS) | % | **0.756** | 0.001 | 0.057 | 0.010 | 0.003 | 0.039 | 0.072 | 0.057 | 0.005 |
| Total free amino acid (TFA) | µg g^-1^ glutamic acid equivalent | **0.725** | 0.018 | 0.002 | 0.001 | 0.015 | 0.110 | 0.128 | 0.000 | 0.001 |

Values in bold correspond for each variable to the factor for which the squared cosine is the largest
